# Sphk1/S1P pathway promotes blood-brain barrier breakdown after intracerebral hemorrhage through inducing Nlrp3-mediated endothelial cell pyroptosis

**DOI:** 10.1038/s41419-024-07310-4

**Published:** 2024-12-23

**Authors:** Mengzhao Feng, Yuan An, Qi Qin, Iat-Hang Fong, Kaiyuan Zhang, Fang Wang, Dengpan Song, Mengyuan Li, Min Yu, Chi-Tai Yeh, Junlei Chang, Fuyou Guo

**Affiliations:** 1 Department of Neurosurgery, The First Affiliated Hospital of Zhengzhou University, Zhengzhou, Henan Province 450000 China; 2 Key Laboratory of Biomedical Imaging Science and System of Chinese Academy of Sciences, Shenzhen Institute of Advanced Technology, Chinese Academy of Sciences, Shenzhen, Guangdong Province 518055 China; 3 Department of Medical Research & Education, Taipei Medical University - Shuang Ho Hospital, New Taipei City, 23561 Taiwan; 4 Continuing Education Program of Food Biotechnology Applications, College of Science and Engineering, National Taitung University, Taitung, 95092 Taiwan

**Keywords:** Blood-brain barrier, Stroke

## Abstract

Intracerebral hemorrhage (ICH) is a severe stroke subtype with high mortality and limited therapeutic options. The blood-brain barrier (BBB) breakdown post-ICH exacerbates secondary brain injury, highlighting the need for targeted therapies to preserve the BBB integrity. We aim to investigate the role of the Sphk1/S1P pathway in BBB breakdown following ICH and to evaluate the therapeutic potential of Sphk1 inhibition in mitigating this breakdown. Using a combination of human patient samples, mouse models of ICH, and in vitro cellular assays, we assessed the expression levels of Sphk1/S1P after ICH and changes of the BBB after ICH. The Sphk1 inhibitor PF543 and siRNAs were utilized to explore the pathway’s impact on BBB integrity and the underlying mechanisms. The results indicate significant upregulation of Sphk1/S1P in the peri-hematomal brain tissue after ICH, which correlates with increased BBB leakage. Pharmacological inhibition of Sphk1 with PF543 attenuates BBB leakage, reduces hematoma volume, and improves neurological outcomes in mice. At the molecular and ultrastructural level, Sphk1 inhibition protects the BBB integrity by preserving tight junction proteins and suppressing endothelial transcytosis. Furthermore, mechanistic studies reveal that Sphk1 promotes Nlrp3-mediated pyroptosis of brain endothelial cells through the ERK1/2 signaling pathway. Taken together, the Sphk1/S1P pathway plays a critical role in ICH-induced BBB breakdown, and its inhibition represents a promising therapeutic strategy for ICH management.

## Introduction

Intracerebral hemorrhage (ICH) is one of the most devastating cerebrovascular diseases that cause death and disability in the world [1]. Although ICH only accounts for 10% to 15% of all types of stroke [2], the mortality rate of ICH within 30 days is as high as 50%, significantly exceeding other types of stroke [3, 4]. Despite major advances in surgical intervention and management of acute ICH, there is no effective treatment to improve the functional outcomes of patients. Therefore, it is urgent to further elucidate the pathophysiological mechanisms of secondary brain injury following ICH to pave ways for the development of therapeutic interventions.

The blood-brain barrier (BBB) is a highly selective biological barrier composed of tightly connected microvascular endothelial cells, pericytes, astrocytes, and neurons, which prevents harmful substances and immune cells from entering the brain tissue from the bloodstream [5, 6]. Among these components, brain microvascular endothelial cells are a crucial part of the BBB [7]. Following ICH, the BBB undergoes extensive and persistent damage, leading to severe edema in the peri-hematomal area, brain displacement and even brain herniation [8]. This exacerbates the secondary brain injury process post-ICH, posing a threat more lethal than the initial hemorrhage. Previous research on the molecular mechanisms of BBB breakdown after ICH has included immune infiltration and inflammatory injury, and involves molecules such as β-Catenin, GSK-3β, Aquaporin-4 (AQP4) and matrix metalloproteinase-9 (MMP9) [9–16]. However, further research is needed to understand the molecular alterations occurring in brain microvascular endothelial cells after ICH.

Sphingosine kinase 1 (Sphk1), a key enzyme in the production of sphingosine-1-phosphate (S1P), has been implicated in neuroinflammation and neurodegeneration following cerebral ischemia [17–23]. The Sphk1/S1P pathway is recognized for its role in inflammatory responses and neuronal injury, with recent research highlighting its upregulation post-ICH and its role in promoting ferroptosis in neurons [24–26]. However, the functional exploration of Sphk1/S1P in BBB disruption following ICH remains lacking. Pyroptosis, an inflammatory cell death pathway, is mediated by Nlrp3 (NOD-like receptor family pyrin domain containing 3), which forms the inflammasome complex and activates Caspase-1, leading to the release of IL-1β and IL-18 [27–32]. While Nlrp3’s role in neuroinflammation and injury post-ICH is established, its direct effects on BBB disruption has not been explored [33–40].

This study investigates the Sphk1/S1P pathway’s role in BBB breakdown post-ICH using both pharmacological inhibition and genetic knockdown. We discovered elevated Sphk1/S1P levels in peri-hematomal brain tissue of ICH patients and mice, with Sphk1 inhibition attenuating BBB leakage, brain edema, and neurological deficits in a mouse model of ICH. Notably, Sphk1 induction in brain endothelial cells post-ICH correlated with increased Nlrp3-mediated pyroptosis. Our findings unveil the Sphk1/S1P pathway as a potential therapeutic target for ICH, offering novel insights into the management of BBB disruption and associated neurological injuries.

## Results

### Sphk1 expression is significantly increased in peri-hematomal endothelial cells after intracerebral hemorrhage (ICH)

**Fig. 1:**
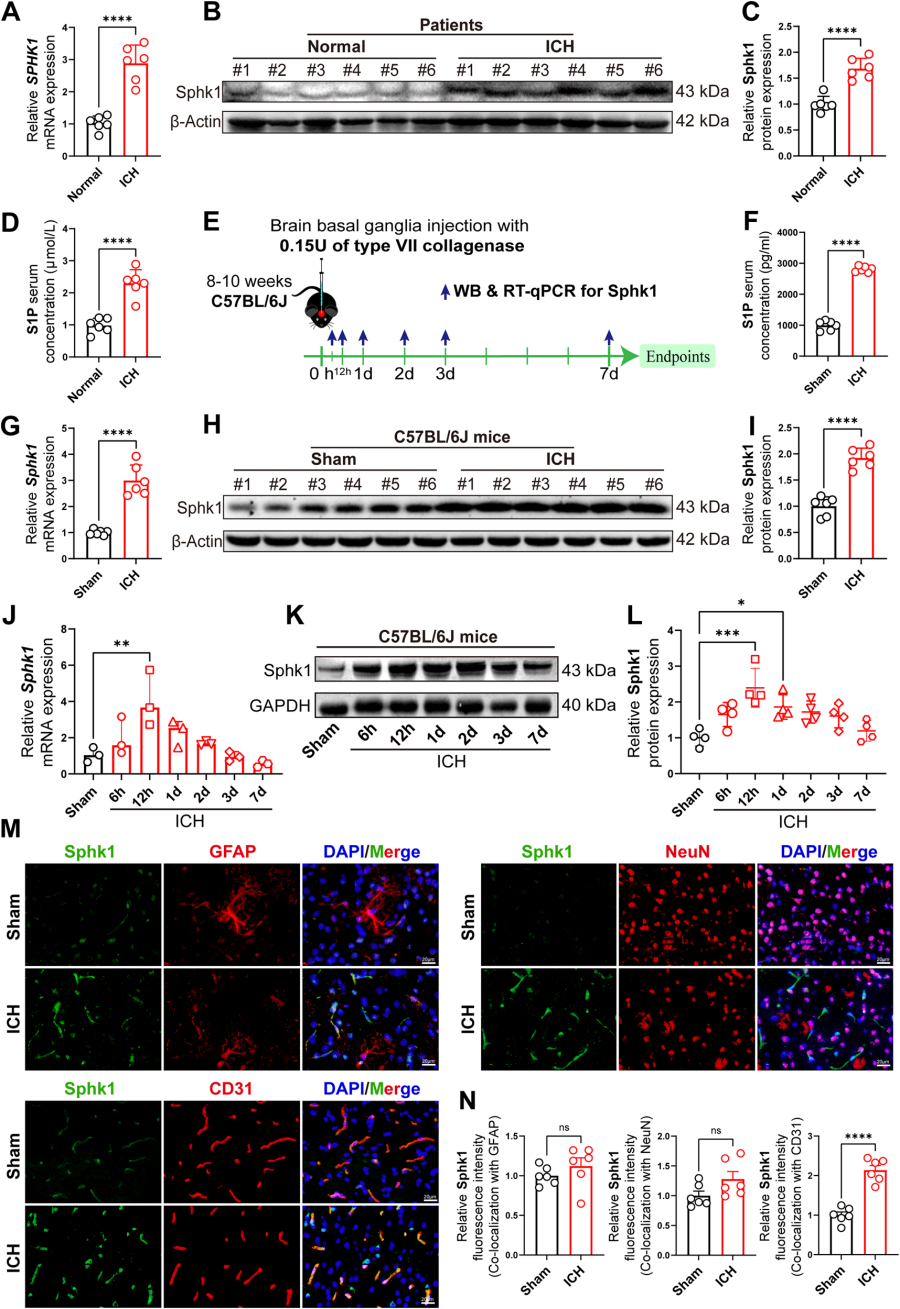
Sphk1 expression is significantly increased in peri-hematomal endothelial cells after intracerebral hemorrhage (ICH). **A** RT-qPCR analysis of peri-hematomal *SPHK1* mRNA expression levels in patients with ICH (*n* = 6 patients/group). **B**, **C** Western blotting analysis of peri-hematomal Sphk1 protein levels in patients with ICH (*n* = 6 patients/group). **D** ELISA analysis of patient serum S1P levels (*n* = 6 patients/group). **E** Schematic for the generation and analysis of the mouse ICH model. **F** ELISA analysis of mouse serum S1P levels at 24 h after ICH (*n* = 6 mice/group). **G** RT-qPCR analysis of peri-hematomal *Sphk1* mRNA expression levels at 24 h after ICH in mice (*n* = 6 mice/group). **H**, **I** Western blotting analysis of peri-hematomal Sphk1 protein levels at 24 h after ICH in mice (*n* = 6 mice/group). **J** RT-qPCR analysis of the time-course changes of *Sphk1* mRNA levels after ICH in mice (*n* = 3 mice/time point, comparisons were conducted between the Sham and ICH mice at different time points). **K**, **L** Western blotting analysis of the time-course changes of Sphk1 protein levels after ICH in mice (*n* = 4 mice/time point, comparisons were conducted between the Sham and ICH mice at different time points). **M** Immunofluorescence staining of peri-hematomal Sphk1 with the astrocyte marker GFAP, neuron marker NeuN and endothelial cell marker CD31 in brain tissue from the sham mice and the ICH mice at 24 h after surgery. Scale bar=20 μm. **N** Quantitative analysis of Sphk1 fluorescence signal density after ICH in mice (*n* = 6 mice/group). Sphk1 fluorescence signal co-localized with respective cellular marker was quantified and normalized by the cellular marker area. Data are expressed as means ± SEM; ns *p* > 0.05, *p < 0.05, **p < 0.01, ***p < 0.001, ****p < 0.0001, two-tailed unpaired Student’s *t*-test except for **J** and (**L**), which used one-way ANOVA and Tukey multiple comparisons test.

To elucidate whether Sphk1/S1P plays a critical role in the course of secondary brain injury after ICH, Sphk1/S1P expression was firstly examined in the peri-hematomal area of patients with ICH. RT-qPCR showed that the expression of *SPHK1* mRNA was significantly increased within 24 h after ICH in patients (Fig. 1A). Western blotting analysis indicated that the Sphk1 protein level was also increased at peri-hematomal area after ICH in humans (Fig. 1B, 1C). Accordingly, the concentrations of serum S1P (Phospholipids synthesized by Sphk1) were significantly elevated in patients after ICH (Fig. 1D). Next, we created a type VII collagenase-based mouse model of ICH to determine whether Sphk1/S1P was also involved in mouse ICH (Fig. 1E). In line with the results of patients, we found that both the mRNA and protein levels of Sphk1 were significantly increased in the peri-hematomal area at 24 h after ICH in mice, along with elevated serum S1P levels (Fig. 1F–I), implying a conservative role of Sphk1/S1P after ICH in mice and humans.

To determine the expression time course of Sphk1, Sphk1 expression in the peri-hematomal brain tissue was measured by RT-qPCR and Western blotting in mice after ICH at multiple time points (Fig. 1E). The results showed that the expression of *Sphk1* mRNA peaked at 12 h after ICH when compared with the sham group (Fig. 1J). Similarly, Sphk1 protein expression was significantly upregulated and peaked at 12 h after ICH in mice (Fig. 1K, L). To determine the cellular location of Sphk1, double immunofluorescence (IF) staining was performed to stain Sphk1 protein with markers for astrocyte, neuron, or endothelial cell, respectively. The result showed that there were a small portion of Sphk1-positive cells colocalized with NeuN signal (Fig. 1M, N), suggesting that some neuronal cells expressed Sphk1 following ICH and thus might contribute to the increase of brain S1P levels. However, the increase of Sphk1 expression in neurons following ICH did not reach statistical significance. Notably, the majority of Sphk1 expression upregulated after ICH was colocalized with vascular endothelial cells, indicating that endothelial cell-derived Sphk1/S1P may act in an autocrine/paracrine manner to regulate the BBB integrity after ICH (Fig. 1M, N, Fig. S1).

### Inhibition of Sphk1 reduces the hematoma volume, brain edema and blood-brain barrier (BBB) leakage after ICH in mice

**Fig. 2:**
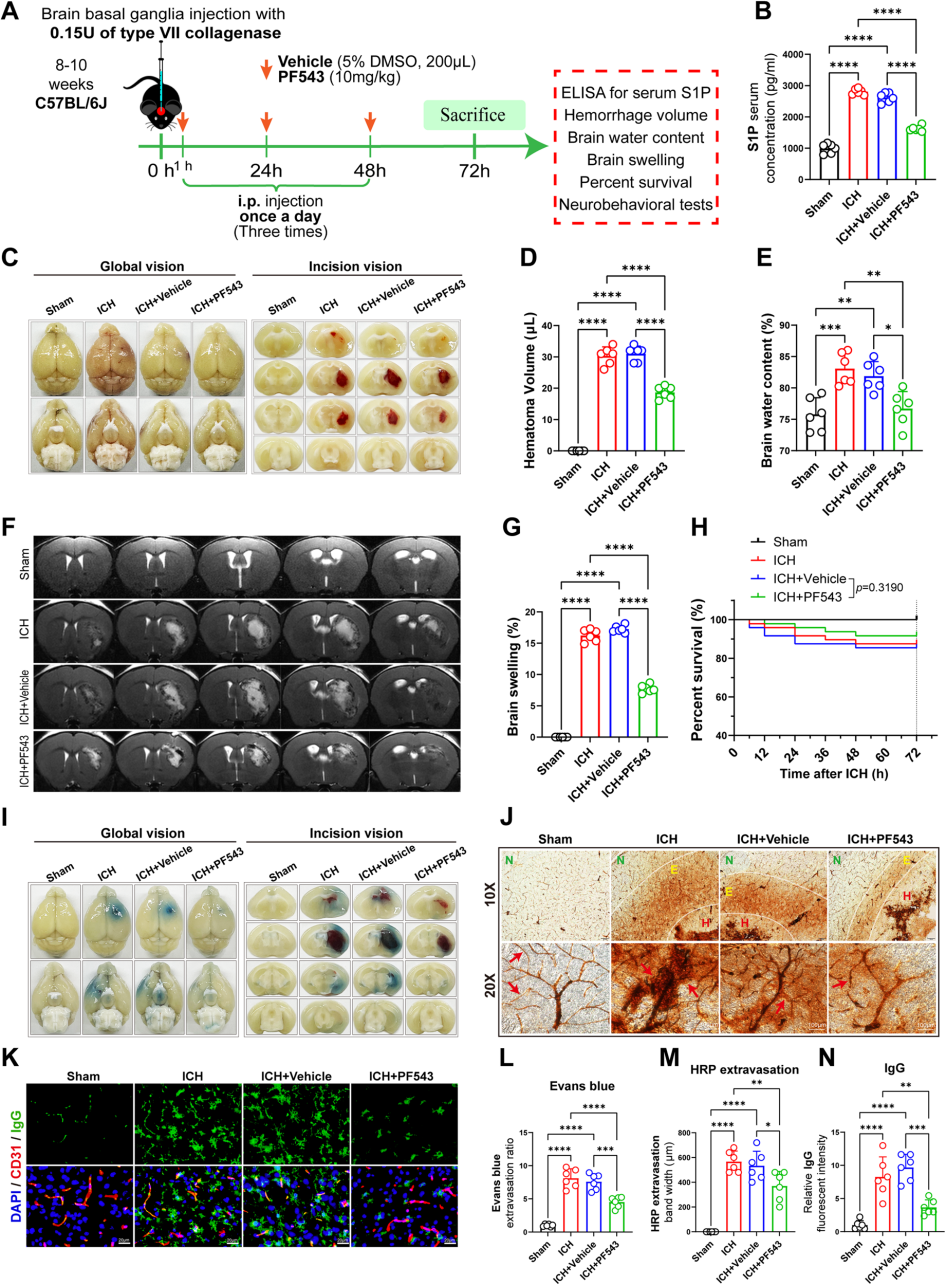
Inhibition of Sphk1 reduces the hematoma volume, brain edema and blood-brain barrier (BBB) leakage after ICH in mice. **A** Patterns of mouse dosing regimen and subsequent molecular biology experiments. **B** ELISA analysis of mice serum S1P concentration (*n* = 6 mice/group). **C** Representative brain images for hematoma volumes. **D** Quantitative analysis of hematoma volume (*n* = 6 mice/group). **E** Quantitative analysis of brain water content (*n* = 6 mice/group). **F** Representative brain magnetic resonance images for brain swelling. **G** Quantitative analysis of brain swelling (*n* = 6 mice/group). **H** Comparisons of survival curves for each group (*n* = 48 mice/group). **I** Evans blue extravasation at peri-hematomal area after ICH. **J** Horse radish peroxidase (HRP) extravasation at peri-hematomal area after ICH (N: Normal. E: Extravasation. H: Hematoma. Arrows indicate varying degrees of HRP extravasation). **K** Immunofluorescence staining of IgG extravasating from endothelial cells at peri-hematomal area after ICH. **L** Quantitative analysis of Evans blue extravasation (*n* = 6 mice/group). **M** Quantitative analysis of HRP extravasation (*n* = 6 mice/group). **N** Quantitative analysis of IgG fluorescence signal density normalized by CD31 area after ICH in mice (*n* = 6 mice/group). Data are expressed as means ± SEM; *p < 0.05, **p < 0.01, ***p < 0.001, ****p < 0.0001, one-way ANOVA and Tukey multiple comparisons test except for H, which used Log-rank (Mantel-Cox) test.

To investigate the role of elevated Sphk1 expression in the secondary brain injury following ICH, we treated the ICH mice with PF543, a highly selective, potent and sphingosine-competitive Sphk1 inhibitor with an IC_50_ of 2 nM and a Ki of 3.6 nM (Fig. 2A) [41]. PF543 was shown to possess >100 folds selectivity for Sphk1 over Sphk2 [41]. Since the expression levels of Sphk1 remained significantly elevated within the first 3 d post-ICH compared to the sham group, we administered the Sphk1 inhibitor PF543 to the ICH mice for 3 consecutive days, once daily, to counteract the full action of Sphk1 post-ICH. As expected, mice treated with PF543 showed significantly decreased serum S1P levels after ICH compared to mice receiving vehicle control, indicating the in vivo efficacy of PF543 treatment (Fig. 2B). Importantly, we found that inhibition of Sphk1/S1P with PF543 substantially decreased the hematoma volume and brain water content in the ICH + PF543 group compared to the ICH + Vehicle group (Fig. 2C-E). Furthermore, T2-weighted 9.4 T MRI analysis showed that the brain swelling was significantly alleviated in ICH + PF543 group mice (Fig. 2F, G). ICH + PF543 group mice have a tendency of high percent survival compared with ICH + Vehicle group (Fig. 2H). Mice in the ICH group and ICH + Vehicle group showed significant motor dysfunction at 3 d after ICH compared with sham group (Fig. S2). Importantly, after treatment with PF543, mice in the ICH + PF543 group showed significant alleviation in motor dysfunction compared to mice in the ICH + Vehicle group (Fig. S2).

Additionally, we performed BBB leakage experiments to further investigate the protective effects of Sphk1 inhibition on the BBB integrity after ICH. Evans blue (EB) extravasation staining showed significantly reduced EB leakage and ameliorated BBB breakdown in the peri-hematomal area of mice in the ICH + PF543 group compared to mice in the ICH + Vehicle group (Fig. 2I–L). Similarly, examination of horse radish peroxidase (HRP) extravasation also showed decreased HRP leakage from brain microvessels in the peri-hematomal area of mice in the ICH + PF543 group compared to mice in the ICH + Vehicle group (Fig. 2J–M). Double IF staining revealed that the endogenous mouse IgG leaked significantly at the peri-hematomal area in the ICH or ICH + Vehicle group compared to the Sham group (Fig. 2K–N). However, the leakage was significantly attenuated by inhibition of Sphk1 (Fig. 2K–N). These data collectively indicate the protective effects of Sphk1 inhibition on BBB breakdown after ICH.

### Inhibition of Sphk1 reduces degradation of tight junction (TJ) proteins after ICH in mice

**Fig. 3:**
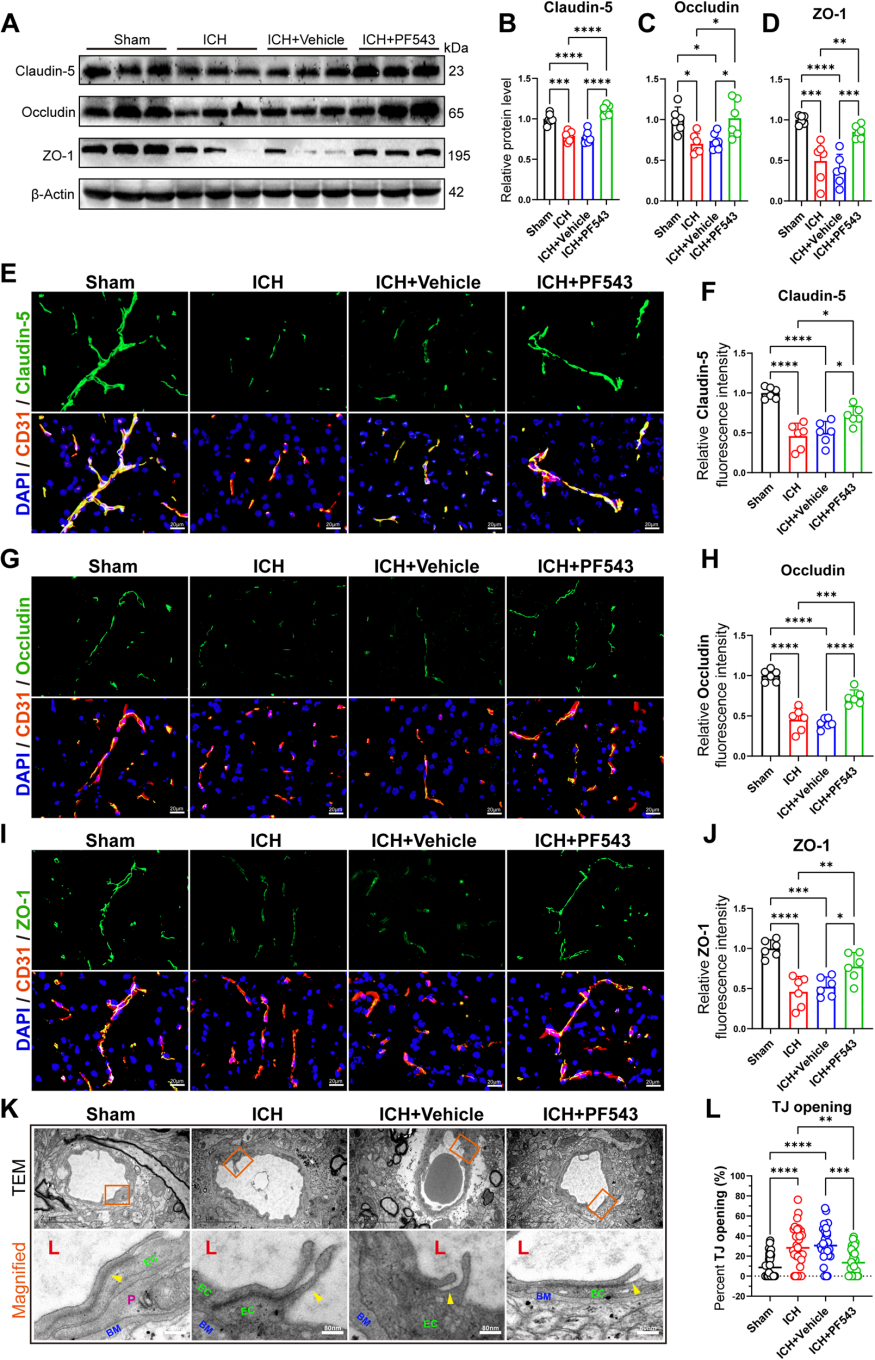
Inhibition of Sphk1 reduces degradation of tight junction (TJ) proteins after ICH in mice. **A**-**D** Western blotting analysis of peri-hematomal TJ proteins levels in mice with ICH (*n* = 6 mice/group). **E** Immunofluorescence staining of Claudin-5 with endothelial cell maker CD31 at peri-hematomal area after ICH in mice. **F** Quantitative analysis of Claudin-5 fluorescence signal density normalized by CD31 area (*n* = 6 mice/group). **G** Immunofluorescence staining of Occludin with CD31 at peri-hematomal area after ICH in mice. **H** Quantitative analysis of Occludin fluorescence signal density normalized by CD31 area (*n* = 6 mice/group). **I** Immunofluorescence staining of ZO-1 with CD31 at peri-hematomal area after ICH in mice. **J** Quantitative analysis of ZO-1 fluorescence signal density normalized by CD31 area (*n* = 6 mice/group). **K** Transmission electron microscope of TJ proteins at peri-hematomal area after ICH in mice (Arrowheads indicate the opening TJ. L: lumen. EC: endothelial cell. BM: basement membrane. P: pericyte.). **L** Quantitative analysis of TJ opening percent. Data are expressed as means ± SEM; *p < 0.05, **p < 0.01, ***p < 0.001, ****p < 0.0001, one-way ANOVA and Tukey multiple comparisons test.

To further define the BBB protective effect of Sphk1 inhibition, we firstly detected the expression levels of intercellular TJ proteins by Western blotting and IF staining. Western blotting results showed that the expressions of Claudin-5, Occludin and ZO-1 were significantly decreased at 3 d after ICH compared with the sham group. However, the expressions of these proteins were significantly increased in the ICH + PF543 group compared with the ICH or ICH + Vehicle group (Fig. 3A–D). Similarly, double IF staining indicated that the relative fluorescence signal density of Claudin-5, Occludin and ZO-1 were significantly decreased at 3 d after ICH compared with the sham group. After treatment with PF543, the relative intensity of these proteins was increased in the ICH + PF543 group compared with the ICH or ICH + Vehicle group (Fig. 3E–J). To further unveil the BBB amelioration, transmission electron microscope (TEM) was used to observe the ultrastructure of the BBB. Representative morphology images observed on TEM revealed that the TJ of peri-hematomal area BBB was obviously opened at 3 d after ICH. However, the opening TJ structure was significantly improved in ICH + PF543 group compared with the ICH or ICH + Vehicle group (Fig. 3K, L).

### Inhibition of Sphk1 decreases endothelial transcytosis after ICH in mice

**Fig. 4:**
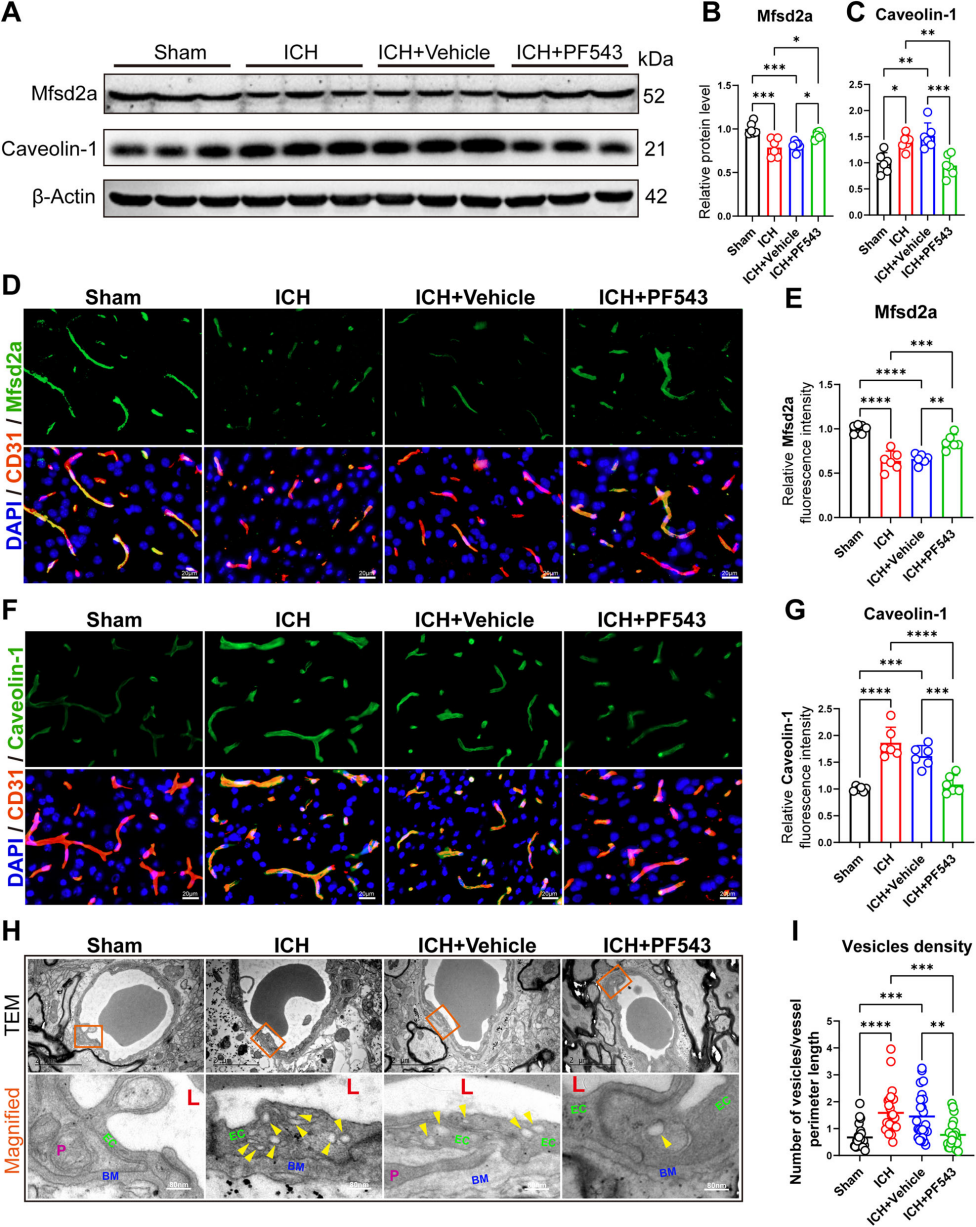
Inhibition of Sphk1 decreases endothelial transcytosis after ICH in mice. **A**-**C** Western blotting analysis of peri-hematomal Mfsd2a/Caveolin-1 proteins levels in mice with ICH (*n* = 6 mice/group). **D** Immunofluorescence staining of Mfsd2a with endothelial cell maker CD31 at peri-hematomal area after ICH in mice. **E** Quantitative analysis of Mfsd2a fluorescence signal density normalized by CD31 area (*n* = 6 mice/group). **F** Immunofluorescence staining of Caveolin-1 with CD31 at peri-hematomal area after ICH in mice. **G** Quantitative analysis of Caveolin-1 fluorescence signal density normalized by CD31 area (*n* = 6 mice/group). **H** Transmission electron microscope of vesicles at peri-hematomal area after ICH in mice (Arrowheads indicated vesicles in endothelial cell. L: lumen. EC: endothelial cell. BM: basement membrane. P: pericyte.). **I** Quantitative analysis of vesicles density normalized by vessel perimeter length. Data are expressed as means ± SEM; *p < 0.05, **p < 0.01, ***p < 0.001, ****p < 0.0001, one-way ANOVA and Tukey multiple comparisons test.

To clarify whether Sphk1 affects transcytosis in BBB endothelial cells, the Mfsd2a (a critical transcytosis inhibitor) and Caveolin-1 (a major caveolae-forming component) proteins were detected by Western blotting and IF staining. Our results showed that the expression of Mfsd2a was decreased at 3 d after ICH compared with the sham group. When administration with Sphk1 inhibitor PF543, the expression of Mfsd2a was increased in the ICH + PF543 group compared with the ICH or ICH + Vehicle group (Fig. 4A, B, D, E). Conversely, our results showed that the expression of Caveolin-1 was increased at 3 d after ICH compared with the sham group. After inhibition of Sphk1, the expression of Caveolin-1 was decreased in the ICH + PF543 group compared with the ICH or ICH + Vehicle group (Fig. 4A, C, F, G). To further determine the transcytosis in endothelial cells, TEM was used to examine the vesicles in the BBB. Representative morphology images revealed that the vesicles in the endothelial cells were obviously increased at 3 d after ICH. Followed by inhibition of Sphk1, vesicles in BBB endothelial cells were significantly reduced in the ICH + PF543 group compared with the ICH or ICH + Vehicle group (Fig. 4H, I).

### Nlrp3 is upregulated in brain endothelial cells after ICH in patients and mice

**Fig. 5:**
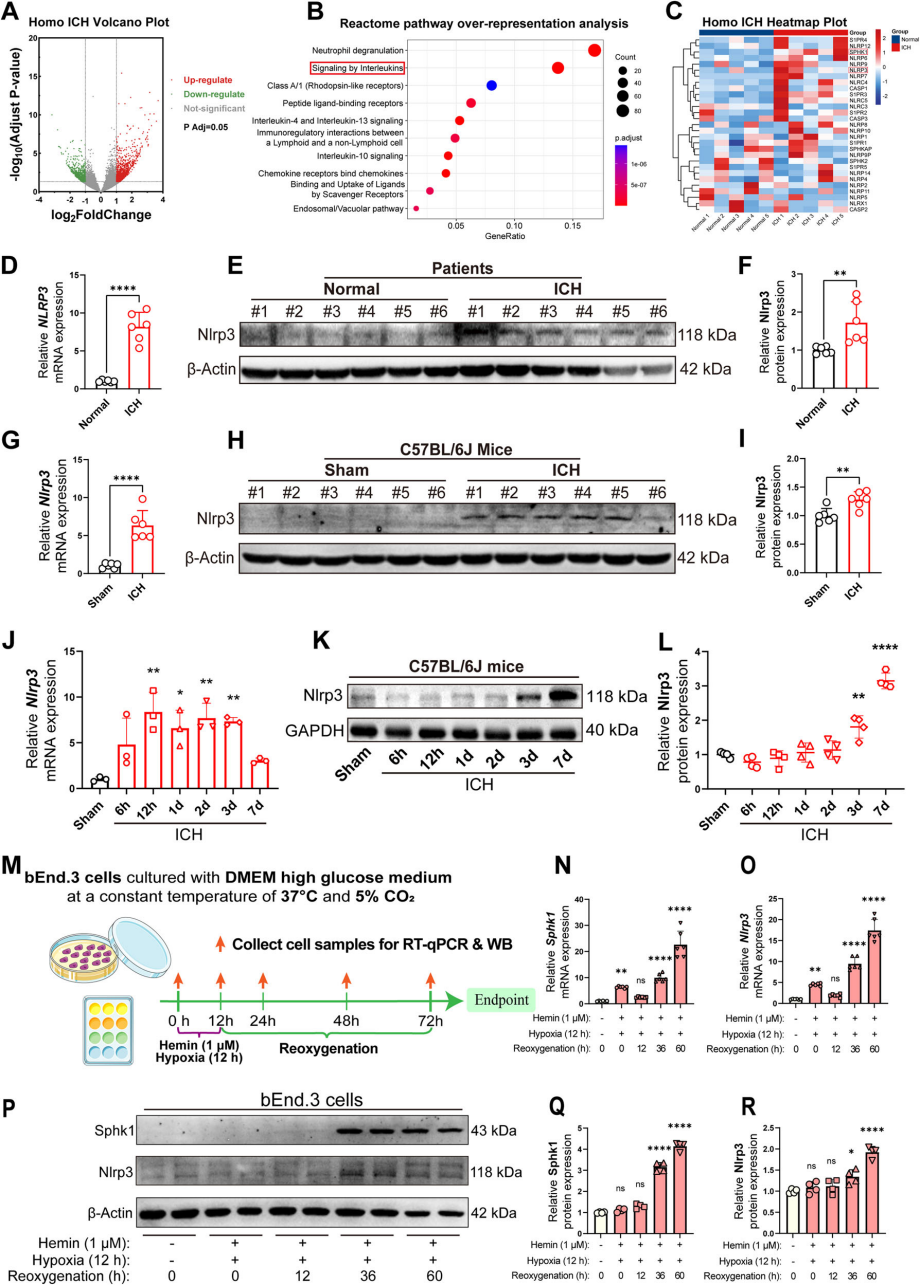
Nlrp3 is upregulated in brain endothelial cells after ICH in patients and mice. **A** Volcano plot analysis of upregulated and downregulated genes after human ICH. **B** Reactome pathway over-representative analysis of upregulated genes in human ICH. **C** Gene heatmaps analysis of the subset of interleukin signaling genes after human ICH. **D** RT-qPCR analysis of peri-hematomal *NLRP3* mRNA expression levels in patients with ICH (*n* = 6 patients/group). **E**, **F** Western blotting analysis of peri-hematomal Nlrp3 protein levels in patients with ICH (*n* = 6 patients/group). **G** RT-qPCR analysis of peri-hematomal *Nlrp3* mRNA expression levels at 3 d after ICH in mice (*n* = 6 mice/group). **H**, **I** Western blotting analysis of peri-hematomal Nlrp3 protein levels at 3 d after ICH in mice (*n* = 6 mice/group). **J** RT-qPCR analysis of the time-course changes of *Nlrp3* mRNA levels after ICH in mice (*n* = 3 mice/time point, comparisons were conducted between the Sham and ICH mice at different time points). **K**, **L** Western blotting analysis of the time-course changes of Nlrp3 protein levels after ICH in mice (*n* = 4 mice/time point, comparisons were conducted between the Sham and ICH mice at different time points). **M** bEnd.3 cellular modeling of ICH and subsequent molecular biology experiments. **N**, **O** RT-qPCR analysis of the time-course changes of *Sphk1* and *Nlrp3* mRNA levels after cellular modeling of ICH in vitro (*n* = 6 replicates/group, comparisons were conducted between the Control and cellular modeling at different time points). **P**, **R** Western blotting analysis of the time-course changes of Sphk1 and Nlrp3 proteins levels after cellular modeling of ICH in vitro (*n* = 4 replicates/group, comparisons were conducted between the Control and cellular modeling at different time points). Data are expressed as means ± SEM; ns *p* > 0.05, *p < 0.05, **p < 0.01, ***p < 0.001, ****p < 0.0001, one-way ANOVA and Tukey multiple comparisons test except for **D**, **F**, **G** and **I**, which used two-tailed unpaired Student’s *t*-test.

To elucidate the role of Sphk1/S1P in BBB damage following ICH, RNA sequencing was performed on human peri-hematomal brain samples. There were 5 peri-hematomal samples and 5 normal brain samples were collected for examination. Comparative analysis identified 1054 upregulated and 887 downregulated genes in the ICH group versus controls (Fig. 5A). Notably, the interleukin signaling pathway, including Nlrp3-mediated pyroptosis, was significantly enriched by reactome pathway over-reprersentation analysis for the 1054 up-regulate genes (Fig. 5B). This was corroborated by increased expression of *SPHK1* (but not *SPHK2*) and *NLRP3* gene in ICH patients (Fig. 5C). Further validation using RT-qPCR and Western blotting confirmed elevated *NLRP3* mRNA and protein levels post-ICH (Fig. 5D–F). In mouse ICH model, Nlrp3 upregulation was observed as early as 12 hours and persisted for up to 3 days post-ICH (Fig. 5G–L). In vitro experiments with mouse brain endothelial cells (bEnd.3) subjected to hemin and hypoxia mimicked the ICH microenvironment. This treatment led to a significant upregulation of *Sphk1* and *Nlrp3* mRNA, with a transient downregulation after 12 hours of reoxygenation, followed by a sustained increase (Fig. 5M–O). Western blotting analysis mirrored these findings at the protein level, with Sphk1 and Nlrp3 expression peaking at 36 hours post-reoxygenation and remaining elevated (Fig. 5P–R).

### Inhibition of Sphk1 suppresses Nlrp3-mediated endothelial cell pyroptosis by ERK1/2 signaling pathway after ICH in mice

**Fig. 6:**
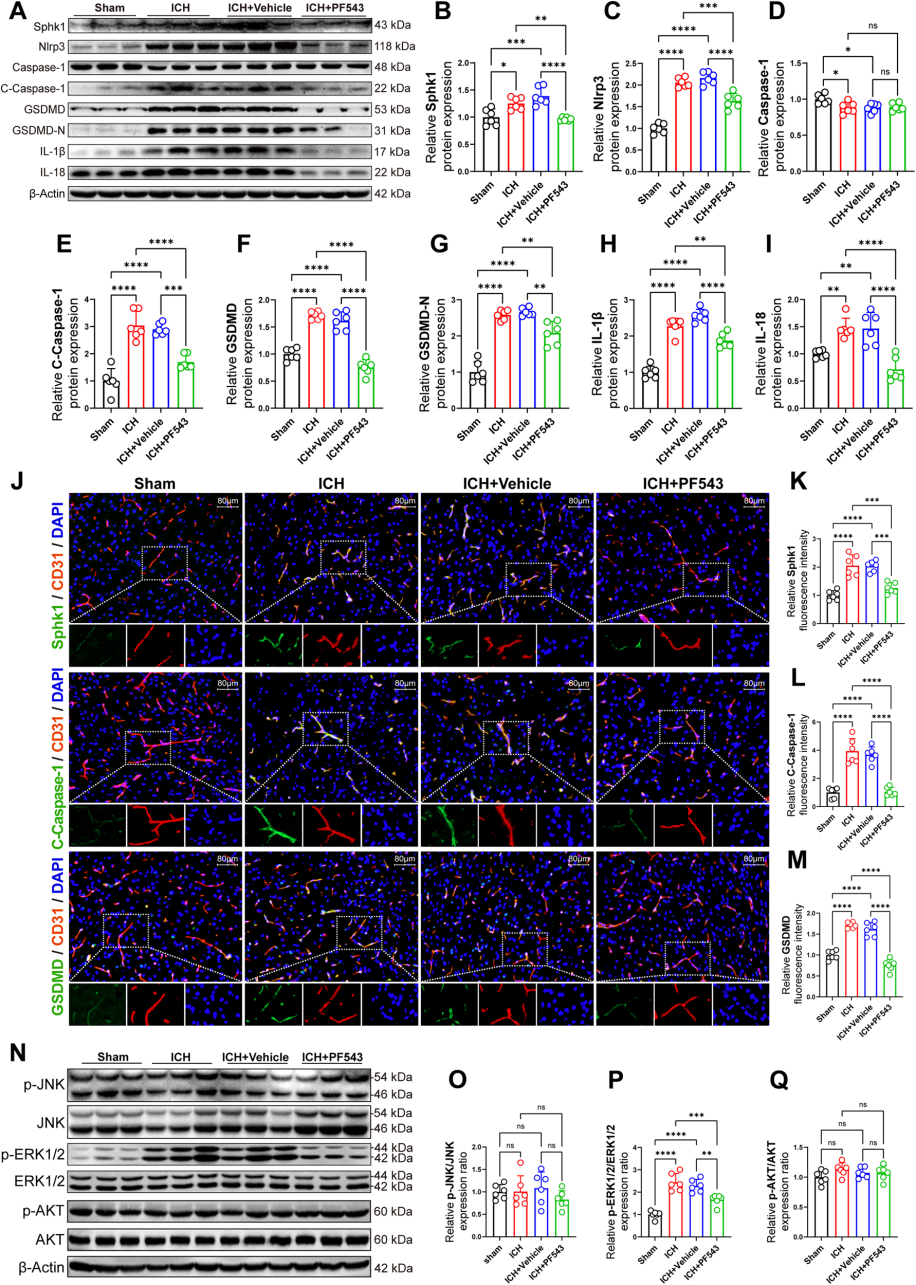
Inhibition of Sphk1 suppresses Nlrp3-mediated endothelial cell pyroptosis by ERK1/2 signaling pathway after ICH in mice. **A**–**I** Western blotting analysis of Sphk1 and Nlrp3-mediated pyroptosis proteins at peri-hematomal area after ICH in mice (*n* = 6 mice/group). **J** Immunofluorescence staining of Sphk1, Cleaved-Caspase-1 (C-Caspase-1) and GSDMD with endothelial cell maker CD31 at peri-hematomal area after ICH in mice. **K**–**M** Quantitative analysis of Sphk1, C-Caspase-1 and GSDMD fluorescence signal density normalized by CD31 area (*n* = 6 mice/group). **N**–**Q** Western blotting analysis of cell signaling pathway proteins at peri-hematomal area after ICH in mice (*n* = 6 mice/group). Data are expressed as means ± SEM; ns *p* > 0.05, *p < 0.05, **p < 0.01, ***p < 0.001, ****p < 0.0001, one-way ANOVA and Tukey multiple comparisons test.

To investigate whether Sphk1/S1P disrupts the BBB via Nlrp3-mediated endothelial cell pyroptosis, we assessed Nlrp3 and pyroptosis-related proteins in PF543-treated mice post-ICH. Western blotting analysis showed increased expression of Sphk1, Nlrp3, cleaved Caspase-1, GSDMD, GSDMD-N, IL-1β, and IL-18 at 3 d post-ICH compared to the sham group, with decreased expression in the ICH + PF543 group (Fig. 6A–I). Double immunofluorescence confirmed co-localization of Sphk1, cleaved Caspase-1, and GSDMD with the endothelial marker CD31, with elevated expression post-ICH, and reduced expression following Sphk1 inhibition with PF543 (Fig. 6J–M). These findings suggest that Sphk1/S1P triggers Nlrp3-mediated endothelial pyroptosis after ICH, and Sphk1 inhibition suppresses this pathway.

Exploring the signaling mechanisms, we focused on the JNK, ERK, and AKT pathways, informed by previous literature [42]. Western blotting revealed no significant changes in p-JNK, total JNK, ERK1/2, p-AKT, and total AKT levels across groups (Fig. 6N, O, and Q). However, a significant increase in p-ERK1/2 levels and the p-ERK1/2 to total ERK1/2 ratio was observed 3 d post-ICH, with a notable decrease in the ICH + PF543 group after Sphk1 inhibition, indicating that Sphk1 regulates ERK1/2 phosphorylation and downstream signaling (Fig. 6P).

### Sphk1 promotes Nlrp3-mediated pyroptosis in brain endothelial cells by ERK1/2 signaling pathway in vitro

**Fig. 7:**
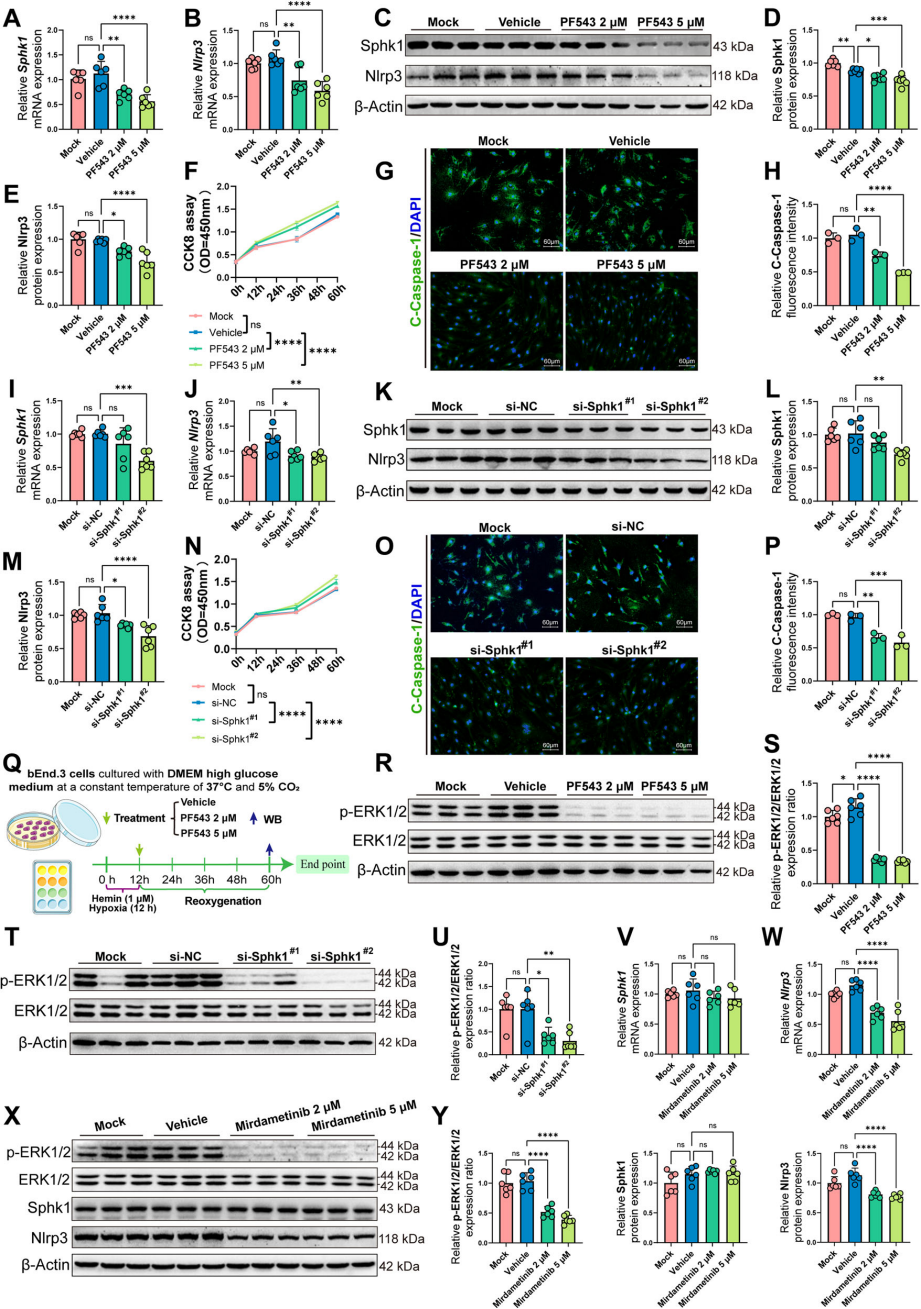
Sphk1 promotes Nlrp3-mediated pyroptosis in brain endothelial cells by ERK1/2 signaling pathway in vitro. **A**, **B** RT-qPCR analysis of the *Sphk1* and *Nlrp3* mRNA levels in bEnd.3 cell model for ICH and PF543 therapy (*n* = 6 replicates/group). **C**–**E** Western blotting analysis of Sphk1 and Nlrp3 proteins levels in bEnd.3 cell model for ICH and PF543 therapy (*n* = 6 replicates/group). **F** CCK8 assay of bEnd.3 cell model for ICH and PF543 therapy (*n* = 3 replicates/group). **G** Immunofluorescence staining of Cleaved-Caspase-1 in bEnd.3 cell model for ICH and PF543 therapy. **H** Quantitative analysis of Cleaved-Caspase-1 fluorescence signal density normalized by DAPI area in bEnd.3 cell model for ICH and PF543 therapy (*n* = 3 replicates/group). **I**, **J** RT-qPCR analysis of the *Sphk1* and *Nlrp3* mRNA levels in bEnd.3 cell model for ICH and si-Sphk1 transfection (*n* = 6 replicates/group). **K**–**M** Western blotting analysis of Sphk1 and Nlrp3 proteins levels in bEnd.3 cell model for ICH and si-Sphk1 transfection (*n* = 6 replicates/group). **N** CCK8 assay of bEnd.3 cell model for ICH and si-Sphk1 transfection (*n* = 3 replicates/group). **O** Immunofluorescence staining of Cleaved-Caspase-1 in bEnd.3 cell model for ICH and si-Sphk1 transfection. **P** Quantitative analysis of Cleaved-Caspase-1 fluorescence signal density normalized by DAPI area in bEnd.3 cell model for ICH and si-Sphk1 transfection (*n* = 3 replicates/group). **Q** Diagram of bEnd.3 cell model for ICH and PF543 therapeutic intervention. **R**, **S** Western blotting analysis of p-ERK1/2 and ERK1/2 in bEnd.3 cell model for ICH and PF543 therapy (*n* = 6 replicates/group). **T**, **U** Western blotting analysis of p-ERK1/2 and ERK1/2 in bEnd.3 cell model for ICH and si-Sphk1 transfection (*n* = 6 replicates/group). **V**, **W** RT-qPCR analysis of the *Sphk1* and *Nlrp3* mRNA levels in bEnd.3 cell model for ICH and Mirdametinib therapy (*n* = 6 replicates/group). **X**,**Y** Western blotting analysis of p-ERK1/2, ERK1/2, Sphk1 and Nlrp3 proteins levels in bEnd.3 cell model for ICH and Mirdametinib therapeutic intervention (*n* = 6 replicates/group). The cells were pre-treated with Hemin (1 μM) and hypoxia for 12 h to mimic ICH in vitro. Data are expressed as means ± SEM; ns *p* > 0.05, *p < 0.05, **p < 0.01, ***p < 0.001, ****p < 0.0001, one-way ANOVA and Tukey multiple comparisons test except for **F** and **N**, which used two-way ANOVA and Tukey multiple comparisons test.

To establish the role of Sphk1 in mediating Nlrp3-dependent pyroptosis in brain endothelial cells, we treated bEnd.3 cells with an Sphk1 inhibitor (PF543) or si-RNA post-ICH simulation (Fig. S3A). PF543 treatment significantly reduced mRNA and protein levels of Sphk1 and Nlrp3 at 2 μM and 5 μM concentrations, compared to the vehicle-treated group (Fig. 7A–E). This was accompanied by enhanced cell proliferation and reduced Cleaved-Caspase-1 expression, indicative of decreased pyroptosis (Fig. 7F–H).

Knockdown of Sphk1 via siRNAs (si-Sphk1^#1^ and si-Sphk1^#2^) following ICH simulation also led to a significant decrease in Sphk1 and Nlrp3 mRNA and protein levels, compared to the negative control group (Fig. 7I–M, S3B). This was associated with increased cell proliferation and a reduction in Cleaved-Caspase-1 expression (Fig. 7N–P).

Further investigation into the ERK1/2 signaling pathway revealed that PF543 and si-Sphk1 treatments significantly reduced the phosphorylation of ERK1/2 (p-ERK1/2) compared to the vehicle-treated group (Fig. 7Q–U, S3C). The use of the ERK1/2 inhibitor Mirdametinib replicated these findings (Fig. S3D), showing reduced p-ERK1/2 levels and decreased Nlrp3 mRNA expression post-ICH simulation (Fig. 7V–Y). These results highlight the regulatory influence of Sphk1 on ERK1/2 phosphorylation and its downstream effects on Nlrp3 expression.

## Discussion

This study delineates the critical role of the Sphk1/S1P pathway in the pathogenesis of BBB disruption following ICH. Our findings reveal a significant upregulation of Sphk1 in peri-hematomal endothelial cells in both human and murine ICH, which was attenuated by Sphk1 inhibition, subsequently reducing hematoma volume, brain edema, and neurological deficits. The modulation of Sphk1 post-ICH not only diminished BBB leakage but also suppressed Nlrp3 expression and pyroptosis in brain endothelial cells, suggesting a regulatory role of Sphk1 in Nlrp3-mediated pyroptosis of brain endothelial cells via the ERK1/2 signaling pathway.

The secondary brain injury post-ICH, including BBB breakdown, neuronal damage, and an exaggerated immune response, often surpass the initial injury in severity [43, 44]. Notably, the cerebral edema that encircles the hematoma post-ICH can precipitate brain displacement and potentially lead to brain herniation, presenting a more severe threat to patient survival [45, 46]. The current therapeutic landscape is limited, with most treatments focusing on symptom alleviation rather than addressing the underlying pathophysiology. Sphk1 serves as a pivotal catalyst in the biosynthesis of S1P, a critical mediator of intracellular signaling pathways. S1P exerts its influence by modulating a diverse array of physiological and pathological processes, including but not limited to inflammation, immune regulation, and angiogenesis [47, 48]. Elevated *Sphk1* gene expression post-ICH has been linked to ferroptosis in neurons, with inhibition strategies, including Sphk1 chemical inhibitors and siRNA, shown to reduce secondary brain injury. This highlights Sphk1 as a therapeutic target for neuroprotection [24]. Additionally, our previous research indicates that S1PR3 inhibition post-ICH can bolster BBB integrity and curb microglial M1 polarization, suggesting a role in neuroinflammation management and neurovascular protection [49]. This study further substantiates Sphk1 as a potential therapeutic target, highlighting its pivotal role in BBB disruption following ICH. Our research identified a notable increase in Sphk1 within peri-hematomal brain tissue post-ICH. While a minority of Sphk1 was expressed within neuronal cells, the major localization of Sphk1 was within vascular endothelial cells in the mouse brain post-ICH, indicating its involvement in the destabilization of tight junction proteins and downregulation of endothelial transcytosis inhibitory proteins. The therapeutic potential of targeting the Sphk1/S1P pathway was evidenced by the reduction in BBB disruption and cerebral edema following treatment with a specific Sphk1 inhibitor. These findings suggest that Sphk1 inhibition may serve as a strategy to protect the neurovascular unit and improve clinical outcomes by preserving BBB integrity.

However, the precise mechanisms by which Sphk1/S1P pathway contributes to BBB breakdown after ICH are not yet fully elucidated. To address this knowledge gap, our research employed RNA sequencing to systematically profile the transcriptome of patients afflicted with ICH. Our RNA sequencing analysis of human ICH patients revealed significant enrichment of the interleukin signaling pathway, implicating a role for Sphk1 in Nlrp3-mediated endothelial cell pyroptosis. Previous studies have shown that abnormally elevated levels of Nlrp3, IL-1β, and IL-18 following ICH are associated with BBB disruption [50]. The Nlrp3 inhibitor glibenclamide and the Raf kinase inhibitor protein (RKIP) have been demonstrated to protect the BBB and reduce cerebral edema by inhibiting Nlrp3 [51]. Additionally, inhibition of the Sphk1/S1P pathway has been shown to reduce pulmonary microvascular leakage by decreasing Nlrp3 expression in macrophages [52]. Suppression of the S1P receptor S1PR2 has also been associated with reduced hepatic inflammation through the downregulation of Nlrp3 [53]. Furthermore, Sphk1 has been implicated in the modulation of Nlrp3 expression via upregulation of SIRT1, playing a role in the inflammatory process of infantile pneumonia [54]. These findings suggest the potential for the Sphk1/S1P pathway to regulate Nlrp3 in various pathological processes. These are further supported by our in vivo and in vitro experiments, which firstly demonstrated that Sphk1 inhibition or knockdown significantly mitigated Nlrp3-mediated endothelial pyroptosis, enhancing BBB integrity and reducing cerebral edema and motor dysfunction in ICH mice.

Additionally, the precise signaling pathway that links the upregulation of Sphk1 to the activation of Nlrp3-mediated pyroptosis following ICH has not yet been fully elucidated. According to previous reports, we screened the AKT, ERK1/2, JNK signaling pathway. Interestingly, we found that Sphk1 modulated Nlrp3 through ERK1/2 signaling pathway. Our findings indicate that Sphk1 inhibition attenuates Nlrp3-mediated endothelial pyroptosis through the modulation of the ERK1/2 pathway, providing a potential mechanistic insight into the therapeutic effects observed. This is corroborated by the neuroprotective effects of the ERK1/2 specific inhibitor Mirdametinib, which mirrored the effects of Sphk1 inhibition by reducing p-ERK1/2 and Nlrp3 levels.

This study presents several key findings that contribute to the understanding of ICH pathophysiology and potential therapeutic targets. Firstly, it reports, for the first time, an increase in Sphk1 expression in endothelial cells of the peri-hematomal brain tissue following ICH. Notably, Sphk1 inhibition is shown to effectively decrease cerebral hematoma volume and edema, reduce BBB permeability, and enhance limb motor function recovery. Secondly, the study reveals that Sphk1 suppression can mitigate BBB damage by preserving TJ proteins and inhibiting endothelial transcytosis. Thirdly, both in vivo and in vitro experiments confirm that Sphk1 inhibition significantly reduces Nlrp3-mediated endothelial pyroptosis, thereby improving endothelial cell viability post-ICH. Lastly, the research demonstrates that Sphk1 inhibition attenuates Nlrp3-mediated endothelial pyroptosis through the reduction of the p-ERK1/2 to total ERK1/2 ratio, providing mechanistic insight into the observed protective effects. These findings collectively advance the therapeutic landscape for ICH by elucidating the critical role of the Sphk1/S1P pathway in endothelial cell function and neurovascular integrity. A prior study has reported that following ICH, Sphk1 is upregulated in neuronal cells, where it contributes to neuronal injury through inducing ferroptosis and thus promotes secondary brain injury post-hemorrhage [24]. In contrast, our study reports that after ICH, Sphk1 is primarily upregulated in the endothelial cells of the BBB and participates in BBB breakdown by inducing endothelial cell pyroptosis. These mechanisms occur in different cell types, suggesting that Sphk1 may exert distinct biological functions in various cell lineages. Moreover, it is plausible that these mechanisms could occur concurrently within the same cell type. Therefore, Sphk1 may also be involved in regulating ferroptosis or other biological processes in brain vascular endothelial cells, which merits further investigation.

The current study acknowledges certain limitations. Firstly, the in vivo mouse ICH model study is performed with the small molecule inhibitor of Sphk1, which lacks tissue/cell specificity and might simultaneously inhibit the activity of Sphk1 in multiple cell types in the brain tissue or the blood. Although we showed that Sphk1 was mainly upregulated in brain endothelial cells post-ICH, we cannot exclude the contribution of other types of cell-derived Sphk1/S1P. Endothelial specific deletion of Sphk1 would further confirm the role of endothelial cell-derived Sphk1/S1P in the BBB breakdown after ICH. Secondly, while the animal studies have corroborated the neuroprotective effects of Sphk1 inhibition post-ICH, including reductions in brain water content and enhancements in limb motor function, it is imperative to conduct further investigations to determine whether these therapeutic benefits are replicable in the clinical setting for patients with ICH.

The findings of the current investigation underscore the role of the Sphk1/S1P pathway in the breakdown of the BBB following ICH, specifically through the mediation of Nlrp3-driven endothelial pyroptosis. Our results indicate that inhibition of the Sphk1/S1P pathway effectively mitigates endothelial cell pyroptosis via the ERK1/2 signaling pathway. These insights position the Sphk1/S1P pathway as a compelling therapeutic target for the treatment of ICH, warranting further exploration in future research endeavors.

## Method details

### Patient selection

Six acute ICH samples and six normal brain samples were collected from the Department of Neurosurgery of the First Affiliated Hospital of Zhengzhou University. The Ethics Committee for human experiments of Zhengzhou University approved all procedures (Approval number: 2021-KY-0156). Informed consent was obtained and approved by the University Review Board. The study was performed in accordance with the Helsinki Declaration. Six patients presenting with acute ICH and basal ganglia hematoma volumes exceeding 40 ml underwent minimally invasive surgery under microscopic guidance. Informed consent was obtained for the collection of peri-hematomal tissue samples. As controls, six normal brain tissue samples were harvested from patients with deep-seated meningiomas during non-functional tissue resection, with written consent explicitly provided for this purpose. No significant differences in general data were found between the two groups.

### Animals

All experimental procedures involving animal study were approved by the Institutional Animal Care and Use Committee (IACUC) of Shenzhen Institute of Advanced Technology, Chinese Academy of Sciences (Approval number: SIAT-IACUC-231116-FMZ-A2385). Adult male C57BL/6 J mice (8–10 weeks, 20–25 g, purchased from Beijing Vital River Laboratory Animal Technology Co., Ltd.) were used in this study. All mice were housed in barrier facilities in a 12 h light/dark cycle with free access to standard mouse diet and water.

#### ICH models

The ICH model was induced by Collagenase in mice as previously studies. Briefly, the mice were anesthetized by inhalation of isoflurane (1.5%, RWD, China) and then placed on a stereotaxic frame. Collagenase VII-S (sterile-filtered, 0.15U in 0.5 μl of sterile saline, Sigma, St. Louis, MO, USA) was injected into the right basal ganglia of mice (coordinates: 0.3 mm anterior, 2.3 mm lateral and 3.8 mm ventral to the bregma) through a Hamilton syringe at a rate of 0.2 μl/min. The needle was remained for 10 min and then withdrawn slowly. The surgical incision was sutured after blocking the skull burr hole with bone wax. The mice were closely monitored until full recovery from anesthesia. Sham surgery was executed following the same procedure without the Collagenase infusion.

### Drug administration

The mice were randomly assigned into four groups: (A) Sham, (B) ICH, (C) ICH+Vehicle, (D) ICH + PF543. ICH model mice received Vehicle (DMSO 10 μl diluted in saline 190 μl) or PF543 (10 mg/kg/d, dissolved in 10 μl DMSO and then diluted in saline 190 μl, Selleck, USA) via intraperitoneal injection at 1 hour after ICH and subsequently received every 24 hours beginning on the second day after ICH, with total 3 days.

### Cell culture experiments

The immortalized mouse brain endothelial cell line bEnd.3 was obtained from American Type Culture Collection (Manassas, VA, USA). bEnd.3 cells were grown in DMEM (Cytiva, China) supplemented with 10% fetal bovine serum (FBS), 100 units/mL of penicillin and 100 μg/mL of streptomycin. bEnd.3 cells were cultured in a constant temperature incubator at 37 °C with 5% CO_2_ and 95% air. All experiments were employed when the density of cells was 90 ~ 100%. Hemin exposure and hypoxia: the media of bEnd.3 cells was added with Hemin (1 μM, sigma, USA), then cell plates were placed in a hypoxia chamber (Billups-Rothenberg Inc., USA), and the air was replaced with mix gas of 95% N_2_ and 5% CO_2_ by flushing, simulating an ICH stimulation in vitro. Cells were exposed to the in vitro ICH condition for 12 h at 37 °C for the following experiments. For pyroptosis inhibition experiments, bEnd.3 cells were treated with PF543 (2 μM, 5 μM), si-Sphk1^#1^ (sequences: 5’-CGCCGUGAAAUUGAGCAAATT-3’(forward) and 5’-UUUGCUCAAUUUCACGGCGTT-3’ (reverse)), si-Sphk1^#2^ (sequences: 5’-GGCAGAGAUAACCUUUAAATT-3’(forward) and 5’-UUUAAAGGUUAUCUCUGCCTT-3’ (reverse)), respectively. Cell samples were collected for RT-qPCR after 24 h inhibition or for Western blotting after 48 h inhibition.

### Neurobehavioral tests

The longa test, bederson’s scale, limb placement, corner turn test and beam walking test were used to evaluate neurological functions as previously described [55, 56]. Longa test was employed to assess forelimb walking and limb motor symmetry, with a maximum score of 4, with higher scores indicating more severe neurological deficits. The bederson’s scale was performed to assess tactile proception, axial sensation and forelimb activity. The limb placement test was used for the selective obstruction of cortical sensory-motor areas including visual induction, tactile induction, and proprioception-induced limb placement response, testing the anterior and lateral limb placement response function in mice. In the corner turn test, the mice were allowed to enter into a 30°corner, the count of turns to the left or right was recorded and repeated 10 times, and the average percentage of left turns was calculated. The beam-walking test was performed by apparatus consisted of a long strip of wood, which was suspended at a height of 60 cm, with the other end attached an enclosed box. After training of crossing the beam to the enclosed box, the mice were placed at the initial 20 cm of the beam, and the time taken to cross the beams and the number of foot slips off the beam were recorded.

### Hemorrhage volume and brain water content

Brain hematoma volume and cerebral edema were measured based on a previous report [57]. In short, in mouse brain slices, the area of the hematoma was multiplied by the thickness of the hematoma volume of each brain slice, and then the sum of all hematoma volumes was calculated. Brain water content was calculated as (wet weight - dry weight)/wet weight × 100%.

### Magnetic resonance imaging (MRI) examination

MRI was used to estimate brain swelling at 72 h after ICH. Mice were anesthetized with 1.5% isoflurane and placed on a scanning bracket. The body temperature, cardiac rhythm and blood oxygen saturation were monitored during scanning. MRI scanning was performed using a 9.4 T MRI scanner (uMR930, United Imaging, China). MRI images were acquired using a fast spin-echo (FSE) sequence to calculate a T2 map. During MRI acquisition, the following parameters were used: Zoom: 1.00, KF: SM, IF: sm, Fix TP: 0, SP: H3.5, field of view (FOV) = 19*19 mm, 0.5 mm slice thickness, Matrix:384×384, TRA > COR 5.0 > SAG - 1.1, WW: 411 and WL: 207. Brain swelling was calculated as: (ipsilateral brain area - contralateral brain area) /ipsilateral brain area × 100%.

### Evans blue extravasation

To prepare 2% Evans blue (EB) dye, EB powder (Sigma-Aldrich, USA) was re-suspended in normal saline. Each mouse was intravenously injected with 100 μl 2% EB dye. After 4 h circulation, mice were deeply anesthetized by inhalation of isoflurane and transcardially perfused with ice PBS to remove the intravascular dye. The brains were divided into ipsilateral hematoma hemispheres and contralateral non-hematoma hemispheres, and then were homogenized in 1 ml of 50% trichloroacetic acid and centrifuged (10,000 rpm, 20 min). After centrifugation, the supernatant was diluted fourth fold with ethanol, then the concentration of EB was measured with a fluorescent reader (Thermo Fisher Scientific, USA) at 620 nm excitation.

### HRP extravasation

HRP type II (0.5 mg/g body weight, Sigma Aldrich, USA) was dissolved in 0.2 ml PBS and then was injected into tail caudal vein. After circulation of 30 min, mouse brains were collected without perfusion and dissected to 3 mm slices and were fixed by 4% paraformaldehyde. Followed dehydration by sucrose, brain samples were further sliced into 30 μm-thick coronal sections and were incubated for 10 min at room temperature with DAB solution (Solarbio, China) to visualize the extravasation of HRP.

### Transmission electron microscopy (TEM)

TEM were performed as previously described [58]. Briefly, mice brain sections were dehydrated in graded ethanol and embedded in epoxy resin. After cut from the block surface, the ultrathin sections (80 nm) were collected on copper grids, stained with uranyl acetate and Reynold’s lead citrate. A JEM-1400Plus transmission electron microscope (JEOL, Tokyo, Japan) was used to scan BBB ultrastructure.

### Western blotting

Western blotting was performed as previously described [59]. Briefly, the mice were deeply anesthetized with isoflurane, followed by intracardial perfusion with iced PBS. The peri-hematomal area of ipsilateral hemispheres were collected and frozen with liquid nitrogen, then stored in a -80°C freezer until use. All collected samples were homogenized in RIPA lysis (Solarbio, China) buffer with a protease inhibitor for 15 min and centrifuged at 12,000 g (4 °C, 15 min), followed by supernatant collection. Protein concentration was measured using a BCA assay (Solarbio, China). Equal amounts of proteins were loaded onto SDS-PAGE gels, followed by electrophoresis and transference on PVDF membranes. The PVDF membrane was blocked with 5% nonfat milk (Thermo Fisher Scientific, USA) for 2 h and incubated at 4 °C overnight with the following primary antibodies: anti-Sphk1 (1:1000, cat. #10670-1-AP, proteintech, China), anti-Claudin-5 (1:1000, cat. #34-1600, Thermo Fisher Scientific, USA), anti-Occludin (1:1000, cat. #33-1500, Thermo Fisher Scientific, USA), anti-ZO-1 (1:1000, cat. #40-2200, Thermo Fisher Scientific, USA), anti-Mfsd2a (1:1000, cat. #ab307690, Abcam, USA), anti-Caveolin-1 (1:2000, cat. #3267S, Cell Signaling Technology, USA), anti-Nlrp3 (1:1000, cat. #15101S, Cell Signaling Technology, USA), anti-Gasdermin D (1:1000, cat. #39754S, Cell Signaling Technology, USA), anti-Cleaved Gasdermin D (1:1000, cat. #10137S, Cell Signaling Technology, USA), anti-Caspase-1 (1:1000, cat. #83383S, Cell Signaling Technology, USA), anti-cleaved-Caspase-1 (1:1000, cat. #89332S, Cell Signaling Technology, USA), anti-IL-1β (1:800, cat. #ab283822, Abcam, USA), anti-IL-18 (1:800, cat. #ab240376, Abcam, USA), anti-ERK1/2 (1:1000, cat. #4695S, Cell Signaling Technology, USA), anti-Phospho-ERK1/2 (1:1000, cat. #4370S, Cell Signaling Technology, USA), anti-Akt (1:1000, cat. #9272S, Cell Signaling Technology, USA), anti-Phospho-Akt (1:1000, cat. #4060S, Cell Signaling Technology, USA), anti-SAPK/JNK (1:1000, cat. #9252S, Cell Signaling Technology, USA), anti-Phospho-SAPK/JNK (1:1000, cat. #4668S, Cell Signaling Technology, USA), anti-β-Actin (1:1500, cat. #66009-1-Ig, proteintech, China), anti-GAPDH (1:2000, cat. #60004-1-Ig, proteintech, China). The next day, the species-specific secondary antibodies (1:2000, cat. #7074P2, cat. #7076P2, Cell Signaling Technology, USA) were used to incubate membranes at room temperature for 1 h. An ECL plus chemiluminescence reagent kit (Amersham bioscience, USA) was selected for immunoblots visualization. Image J (NIH, Bethesda, USA) was used to quantify the density of band, and the results were normalized to β-Actin or GAPDH.

### Immunofluorescence staining

Immunofluorescence staining was performed as previously described [60]. Briefly, mice were perfused intracardially with ice-cold PBS, and brain samples were collected and followed by fixation in 4% paraformaldehyde overnight at 4 °C and then dehydrated sequentially by 15% and 30% sucrose solutions. For the immunofluorescence staining with fixative-sensitive antibodies, namely antibodies for ZO-1, Occludin, and Claudin-5, fresh brain tissues were directly embedded in OCT. After being frozen in OCT, the brain samples were sliced into 10 μm-thick coronal sections by a freezing microtome (Leica CM 1950, Germany). Followed by washing three times with PBST for 10 min per time, the brain sections were incubated with 5% goat serum at room temperature for 1 h and then incubated at 4 °C overnight with the following primary antibodies: anti-Sphk1 (1:50, cat. #10670-1-AP, proteintech, China), anti-Iba-1 (1:200, cat. #GB15105-100, Servicebio, China), anti-GFAP (1:1000, cat. #PA1-10004, Thermo Fisher Scientific, USA), anti-NeuN (1:500, cat. #94403, Cell Signaling Technology, USA), anti-CD31 (1:500, cat. #MAB1398Z, Merck, Germany), anti-Claudin-5 (1:100, cat. #34-1600, Thermo Fisher Scientific, USA), anti-Occludin (1:100, cat. #33-1500, Thermo Fisher Scientific, USA), anti-ZO-1 (1:100, cat. #21773-1-AP, proteintech, China), anti-Mfsd2a (1:1000, a homemade rabbit polyclonal antibody validated in our previous study) [61], anti-Cavoelin-1 (1:1000, cat. #3267S, Cell Signaling Technology, USA), anti-cleaved-Caspase-1 (1:100, cat. #89332S, Cell Signaling Technology, USA), anti-GSDMD (1:100, cat. #39754S, Cell Signaling Technology, USA). In the following day, the brain slices were incubated with fluorescence-conjugated secondary antibodies (1:500, Jackson Immuno Research, USA) for 1 h at room temperature. Slides were mounted in anti-fade reagent with DAPI (cat. #S2110, Solarbio, China) and imaged with a fluorescence microscope (Zeiss Axio Imager Z2 with Apotome.2).

### Enzyme-linked immunosorbent assay (ELISA)

Enzyme-linked immunosorbent assay was performed according to manufacturer’s instructions. Serum from humans and mice were obtained by centrifugation of blood at 4°C for 20 min, and the supernatant was used for subsequent experiments. The levels of S1P were measured using commercial ELISA kits (cat. #RXJ106471H, Ruixin, China).

### Real-time qPCR (RT-qPCR)

Total mRNA was extracted from tissues and cells with the kits according to the manufacturer’s instructions (Vazyme, China), followed by cDNA conversion using a high-capacity cDNA reverse transcription kit (Vazyme, China). The mRNA expression of each gene was analyzed by RT-qPCR using SYBR Green Mix (Vazyme, China) and the LightCycler 96 instrument (Roche, Swiss). Data were normalized to internal control gene β-Actin. The following primer sequences were used: Human: *SPHK1* forward primer: TCTGCTTGGTCCAATGTGCAA, *SPHK1* reverse primer: GGAACAGTTCGTGTCATCCTC. *NLRP3* forward primer: GATCTTCGCTGCGATCAACAG, *NLRP3* reverse primer: CGTGCATTATCTGAACCCCAC. *ACTB* forward primer: GCTGCATTTAGTGGCCTCATT, *ACTB* reverse primer: GCAAGGCATAACCTGATGTGG. Mouse: *Sphk1* forward primer: GCAACGTGGAATCACCACTGA, *Sphk1* reverse primer: CAGCCAGTAGTCTGTGGACTC. *Nlrp3* forward primer: ATTACCCGCCCGAGAAAGG, *Nlrp3* reverse primer: TCGCAGCAAAGATCCACACAG. *Actb* forward primer: ATGACCCAAGCCGAGAAGG, *Actb* reverse primer: CGGCCAAGTCTTAGAGTTGTTG.

### RNA-seq analysis

Human peri-hematomal brain tissue that was confirmed by computed tomography and intraoperation were used for RNA-sequencing. According to a previous report [62], RNA extraction and quantification were executed, followed by sequencing of the total RNA profile with HiSeq 4000 (Illumina, USA).

### Statistical analysis

Data analysis was employed by GraphPad Prism (Graph Pad Software, USA). When calculating the relative fluorescence intensity within the co-localization area of different cellular markers, the Region of Interest (ROI) selection function in ImageJ was utilized. First, the ROI for the cellular marker was defined, and then the relative fluorescence intensity of the protein of interest within the ROI was calculated and normalized to the area of respective cellular marker. All data were expressed as the mean and standard error of the mean (mea*n* ± SEM). Difference significance of two groups was assessed using Student’s *t*-test or non-parametric Mann-Whitney test. For comparisons of three or more groups, one-way ANOVA followed by Tukey’s post hoc test was used to compare the difference significance among multi groups. Statistical significance was defined as *p* < 0.05.

## Supplementary information

aj-Checklist

Supplementary figures

Supplementary figure legends

Original WB

## Data Availability

The supporting data for the findings of this study can be obtained from the corresponding author upon reasonable request.
