# Supplementary figures and images for: Sphk1/S1P pathway promotes blood-brain barrier breakdown after intracerebral hemorrhage through inducing Nlrp3-mediated endothelial cell pyroptosis
Source: Cell Death Dis. 2024 Dec 23;15(12):926. doi: 10.1038/s41419-024-07310-4 (PMC11666774; doi:10.1038/s41419-024-07310-4)

**Supplementary figures**

**Figure S1.**

**
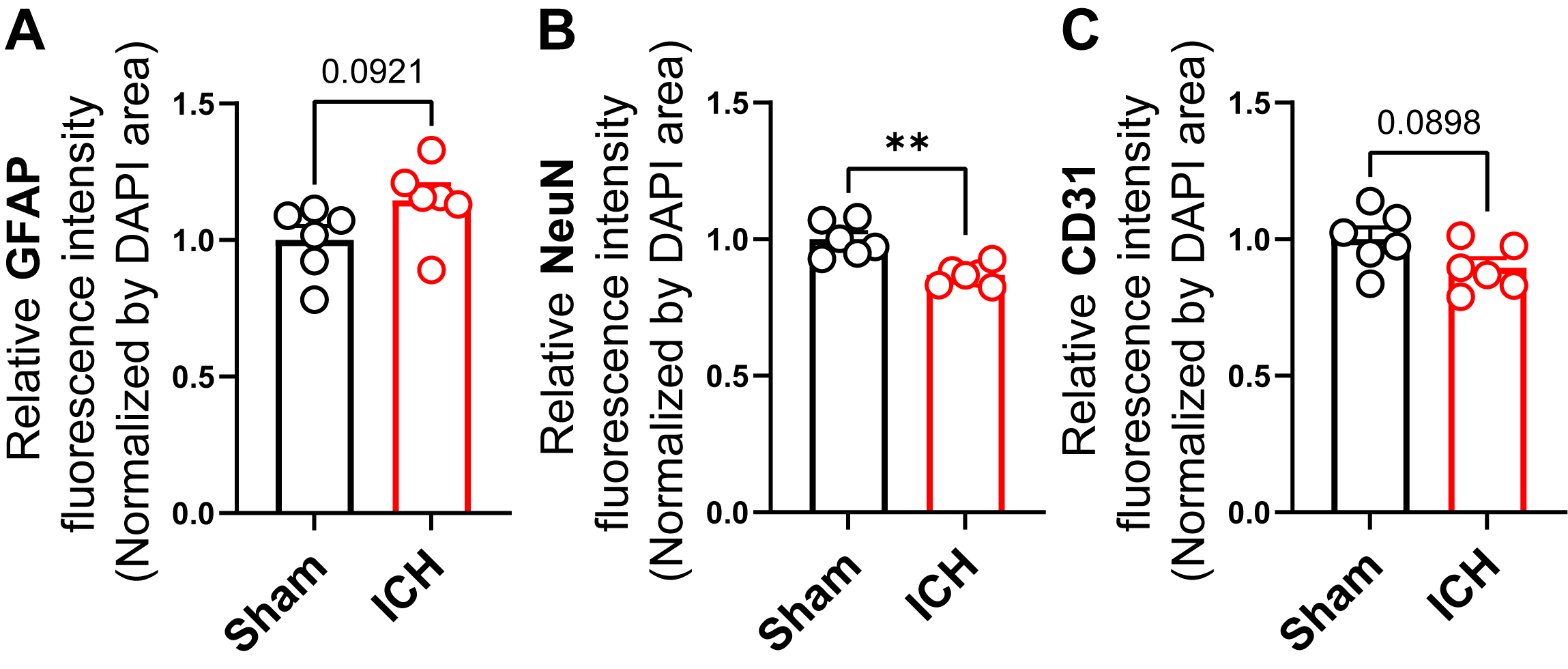
**

**Figure S2.**

**
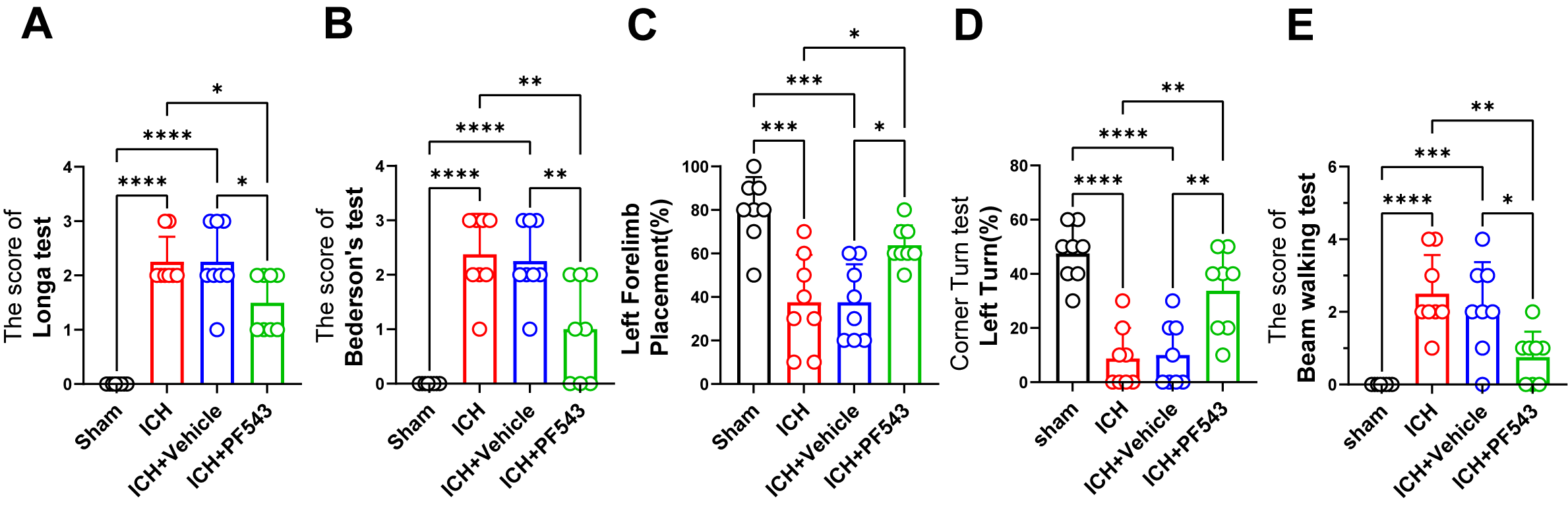
**

**Figure S3.**


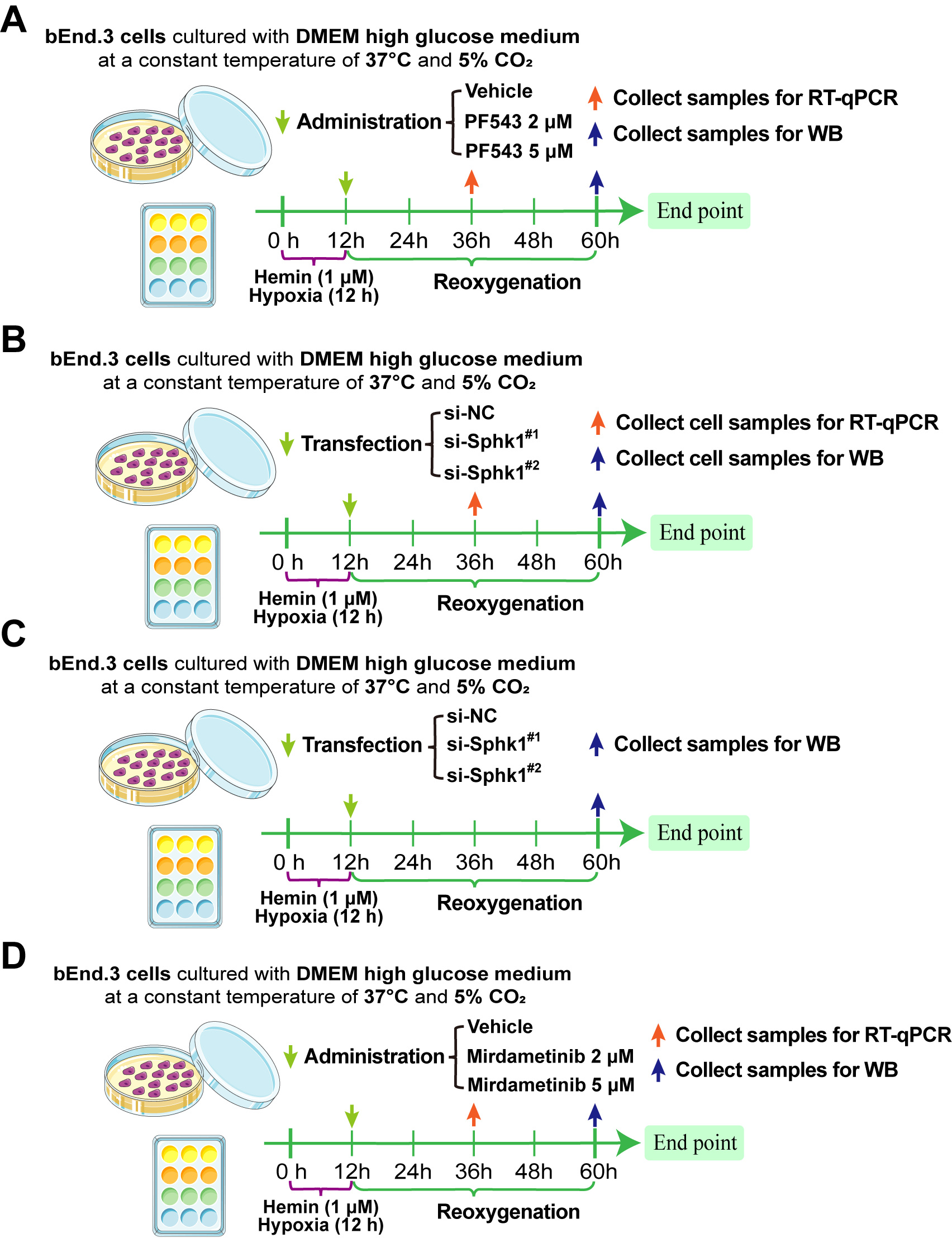

Supplement: Supplementary file 2 — Supplementary figures [file 41419_2024_7310_MOESM2_ESM.doc]
