# Supplementary material for: Sphk1/S1P pathway promotes blood-brain barrier breakdown after intracerebral hemorrhage through inducing Nlrp3-mediated endothelial cell pyroptosis
Source: Cell Death Dis. 2024 Dec 23;15(12):926. doi: 10.1038/s41419-024-07310-4 (PMC11666774; doi:10.1038/s41419-024-07310-4)
Supplement: Supplementary file 3 — Supplementary figure legends [file 41419_2024_7310_MOESM3_ESM.docx]

**Supplemental figure legends:**

**Figure S1:** NeuN expression is reduced in neurons at 24 h after ICH in mice. (A) Quantitative analysis of GFAP fluorescence signal density normalized by DAPI area (n=6 mice/group). (B) Quantitative analysis of NeuN fluorescence signal density normalized by DAPI area (n=6 mice/group). (C) Quantitative analysis of CD31 fluorescence signal density normalized by DAPI area (n=6 mice/group). Data are expressed as means ± SEM; ***p* < 0.01, two-tailed unpaired Student’s *t* test.

**Figure S2:** Inhibition of Sphk1 reduces motor function impairment after ICH in mice. (A-E) Neurobehavioral scores for motor function (n=6 mice/group). Data are expressed as means ± SEM; **p* < 0.05, ***p* < 0.01, ****p* < 0.001, *****p* < 0.0001, one-way ANOVA and Tukey multiple comparisons test.

**Figure S3:** Diagram of bEnd.3 cell model for ICH and multiple treatment. (A) Diagram of bEnd.3 cell model for ICH and PF543 therapeutic intervention. (B) Diagram of bEnd.3 cell model for ICH and small interfering RNA transfection. (C) Diagram of bEnd.3 cell model for ICH and si-Sphk1 transfection. (D) Diagram of bEnd.3 cell model for ICH and Mirdametinib therapeutic intervention.
