# Supplementary material for: Sphk1/S1P pathway promotes blood-brain barrier breakdown after intracerebral hemorrhage through inducing Nlrp3-mediated endothelial cell pyroptosis
Source: Cell Death Dis. 2024 Dec 23;15(12):926. doi: 10.1038/s41419-024-07310-4 (PMC11666774; doi:10.1038/s41419-024-07310-4)

Full uncropped blots images for Figure 1

Figure-1B-Sphk1

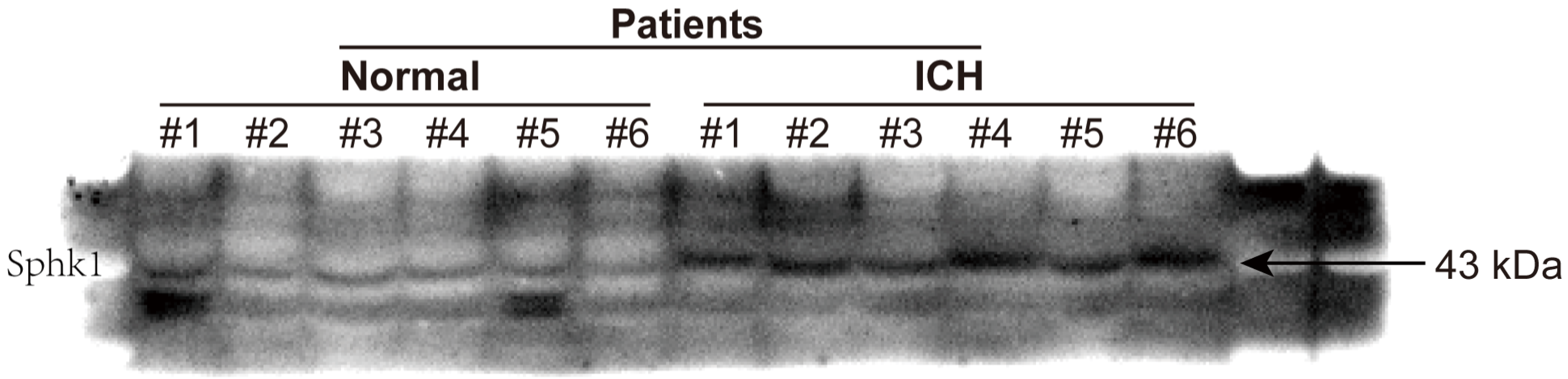

Figure-1B-β-Actin

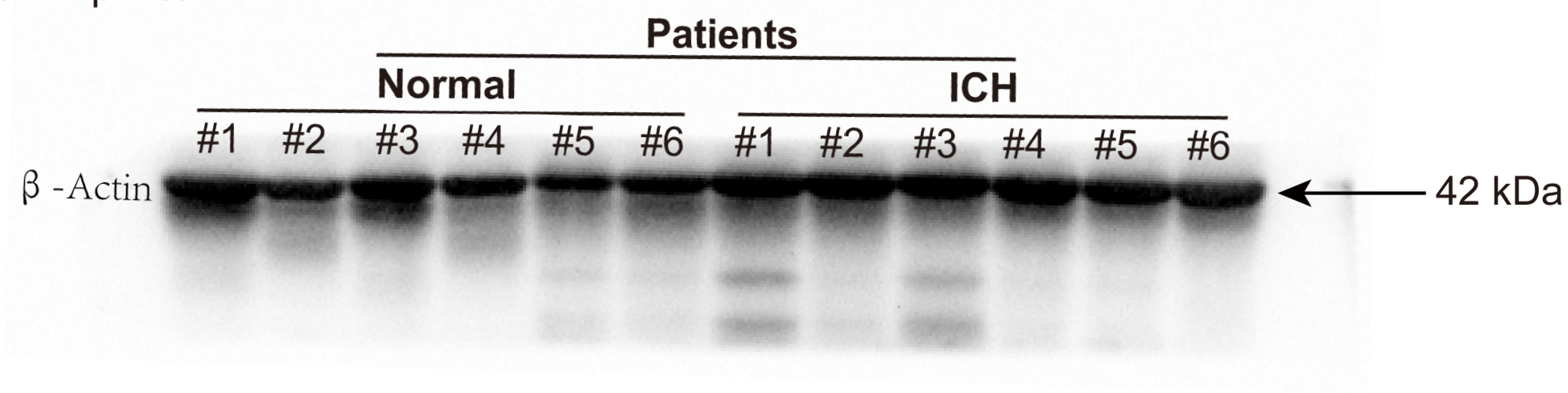

Figure-1H-Sphk1

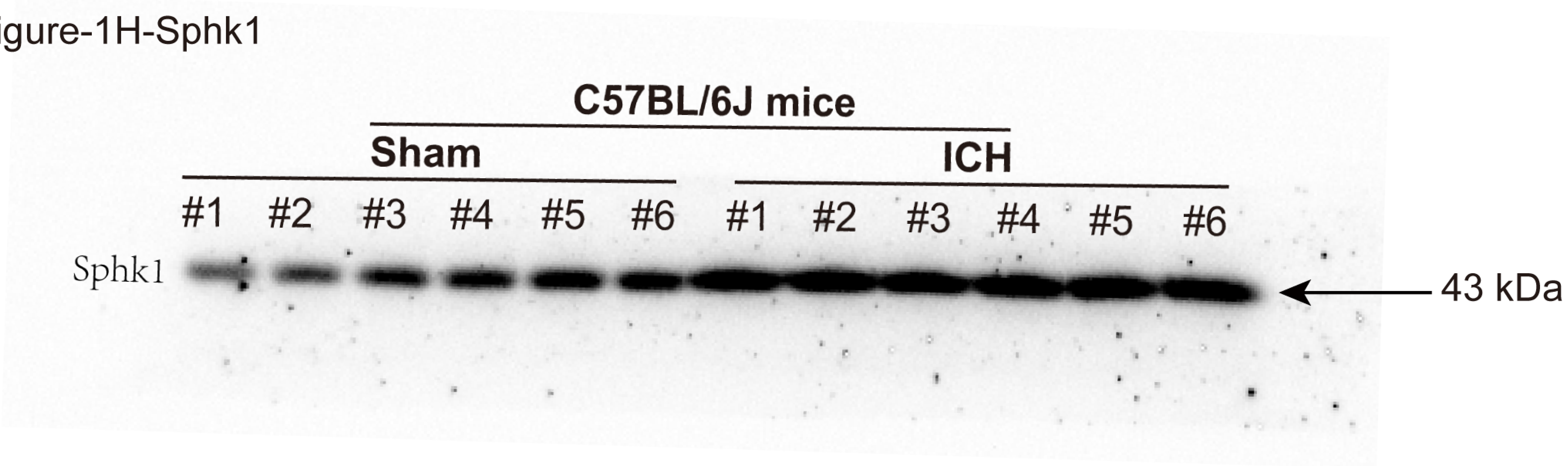

Figure-1H-β-Actin

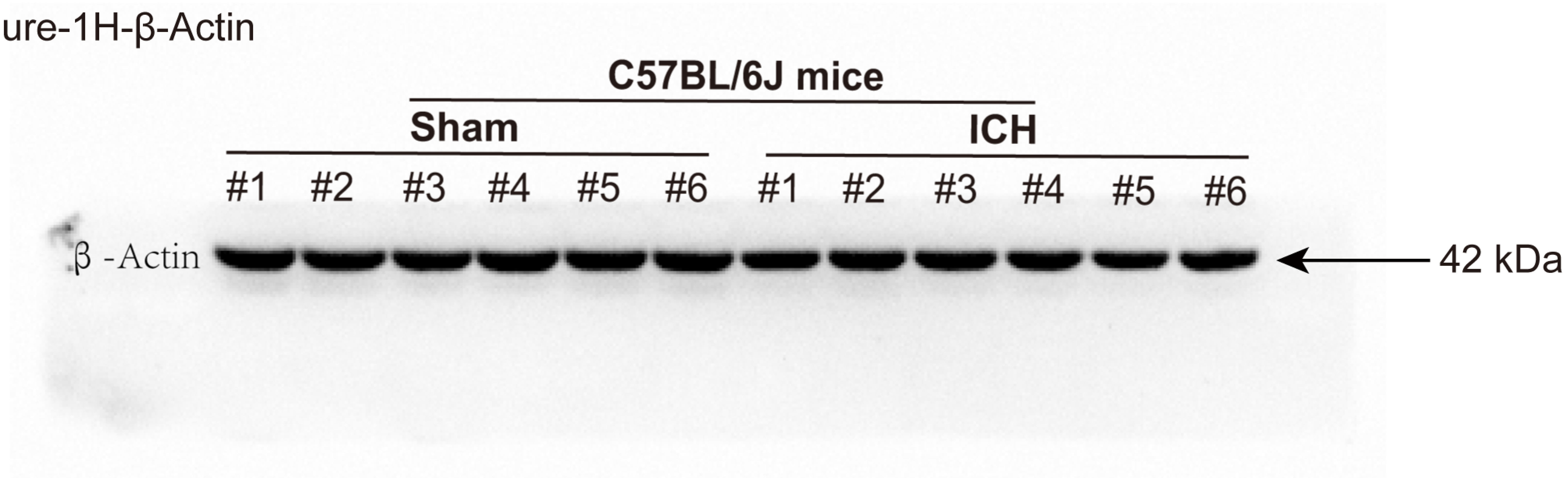

Figure-1K-Sphk1

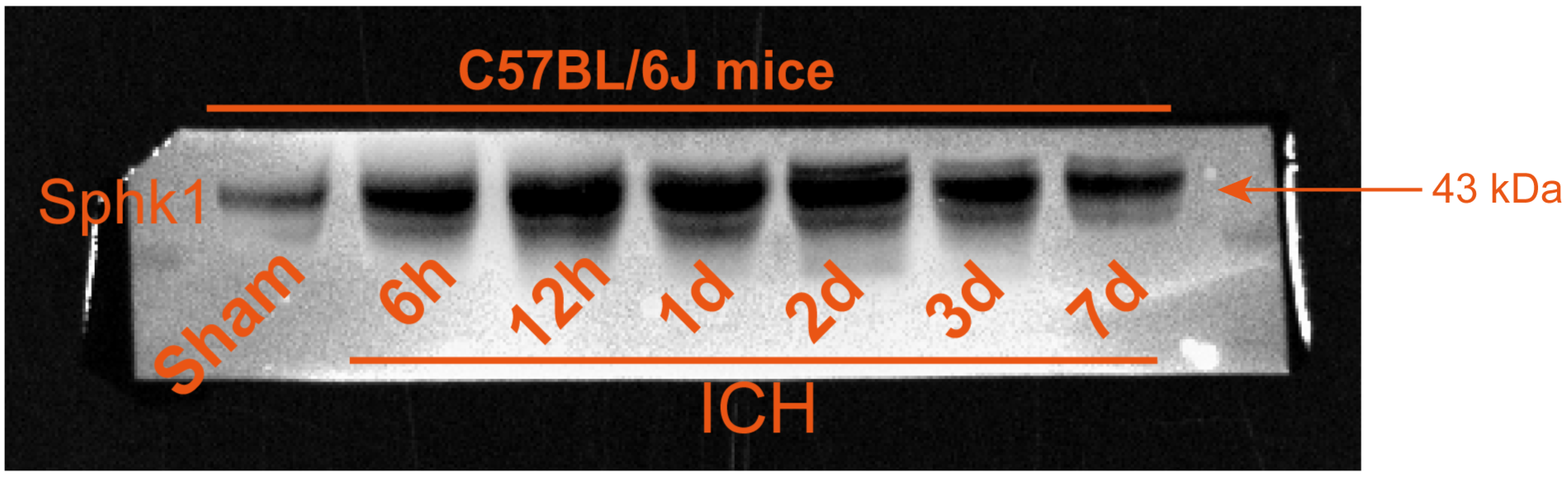

Figure-1K-GAPDH

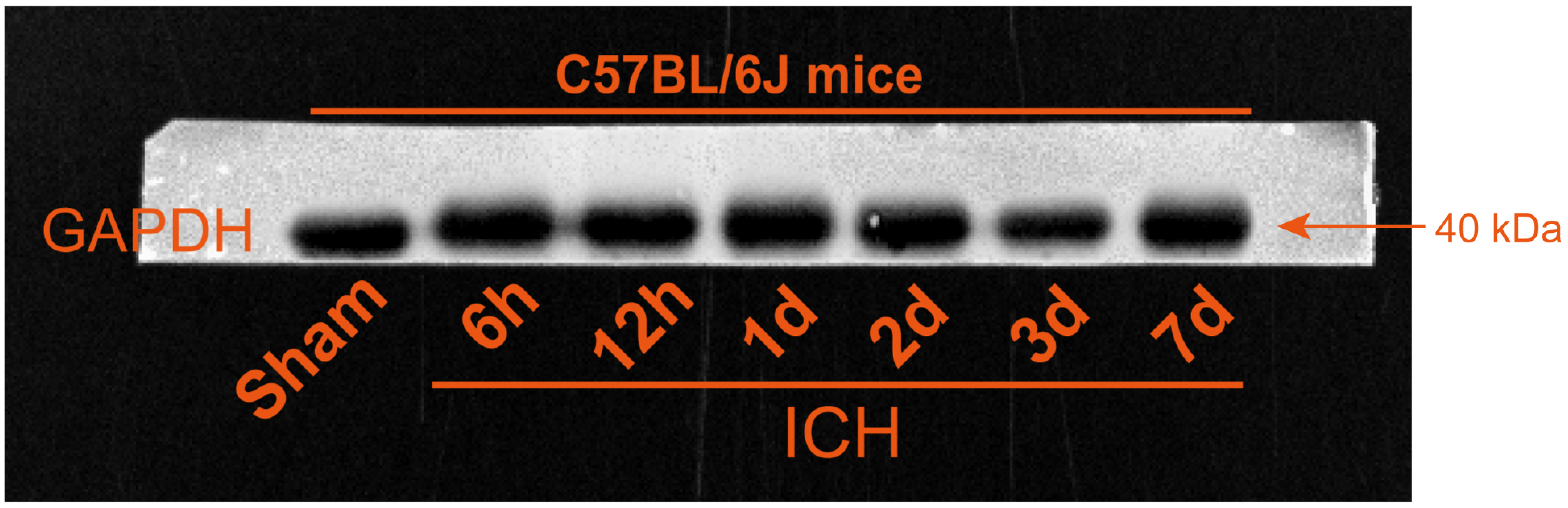

Full uncropped blots images for Figure 3

Figure-3A-Claudin-5

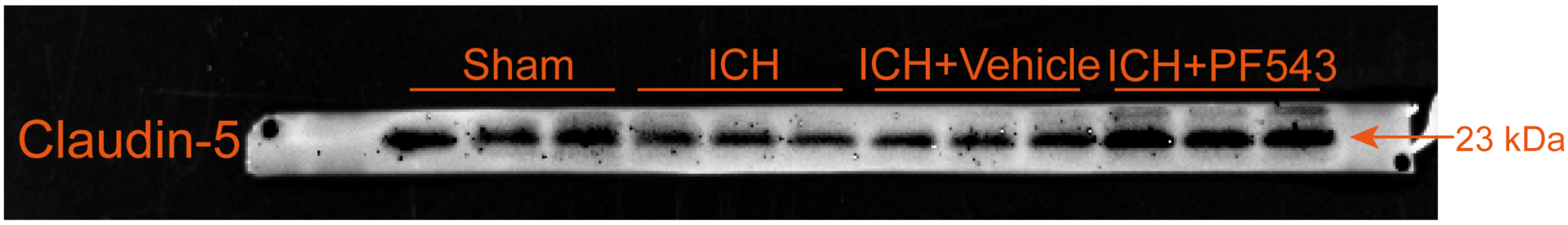

Figure-3A-Occludin

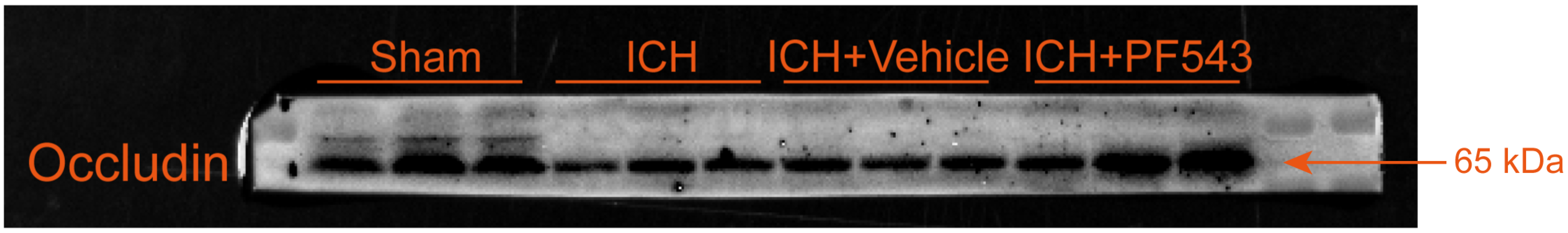

Figure-3A-ZO-1

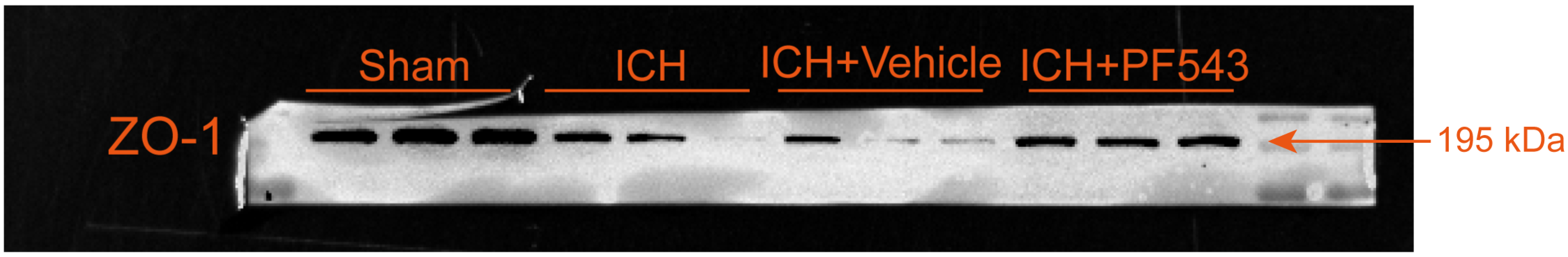

Figure-3A-β-Actin

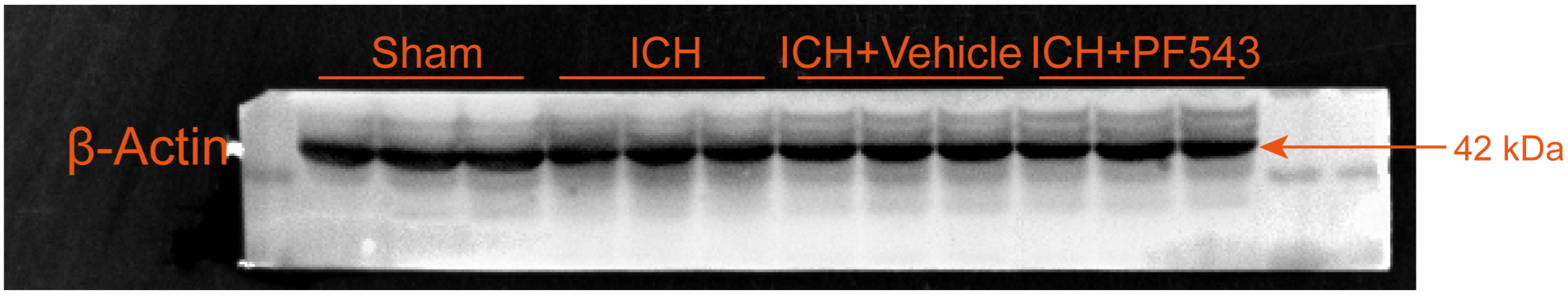

Full uncropped blots images for Figure 4

Figure-4A-Mfsd2a

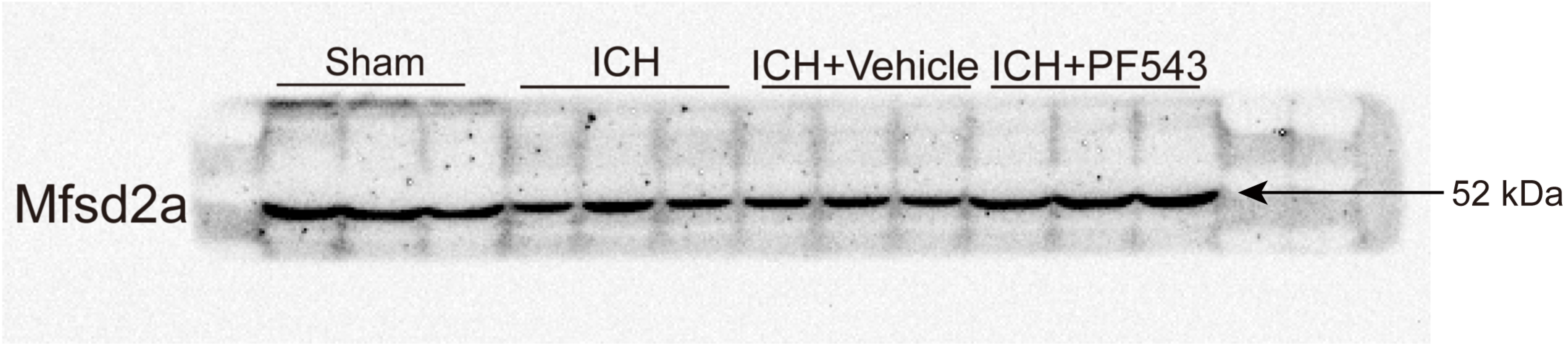

Figure-4A-Caveolin-1

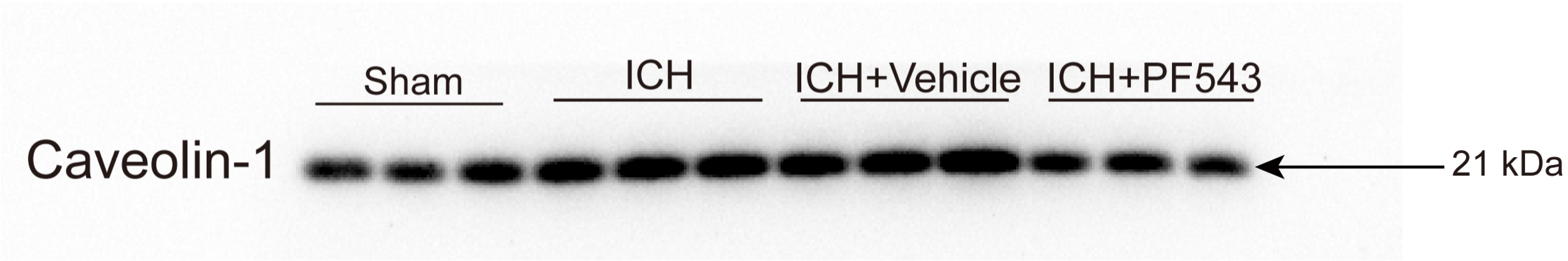

Figure-4A-β-Actin

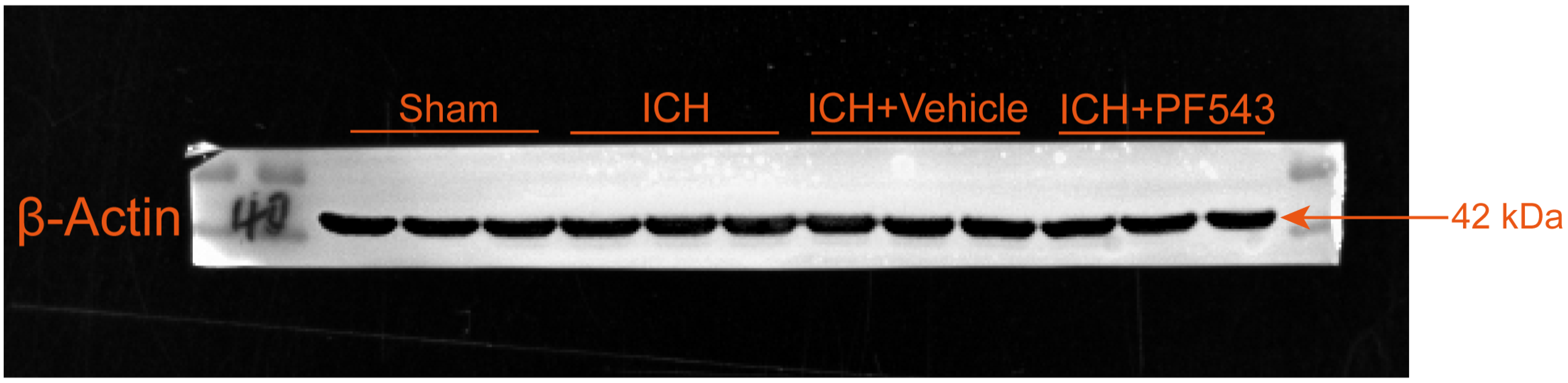

Full uncropped blots images for Figure 5

Figure-5E-Nlrp3

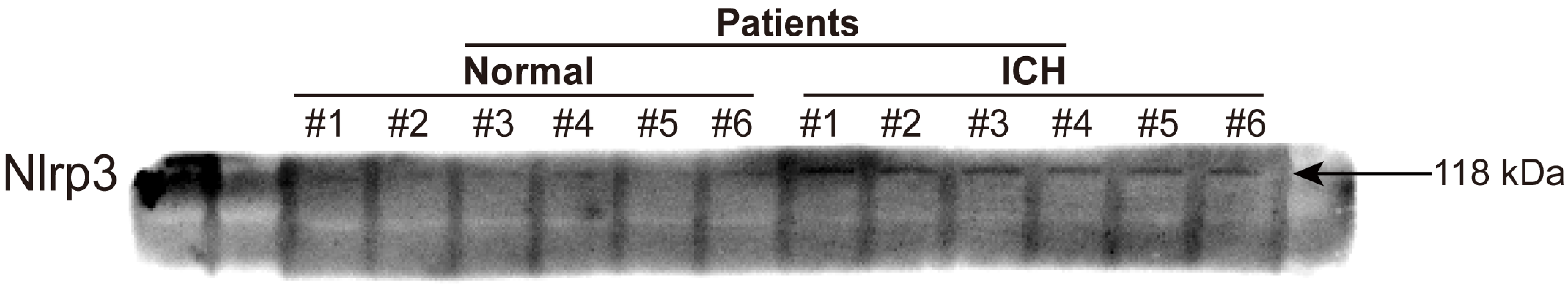

Figure-5E-β-Actin

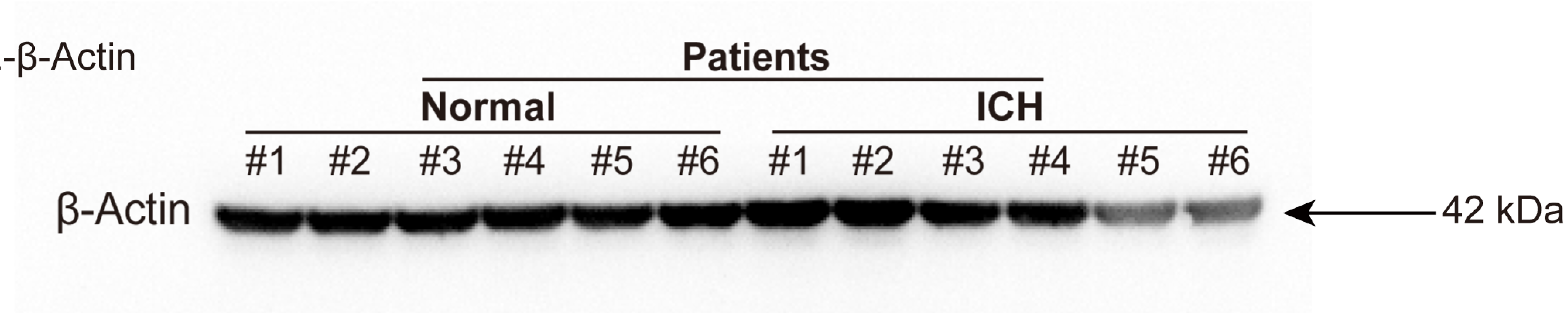

Figure-5H-Nlrp3

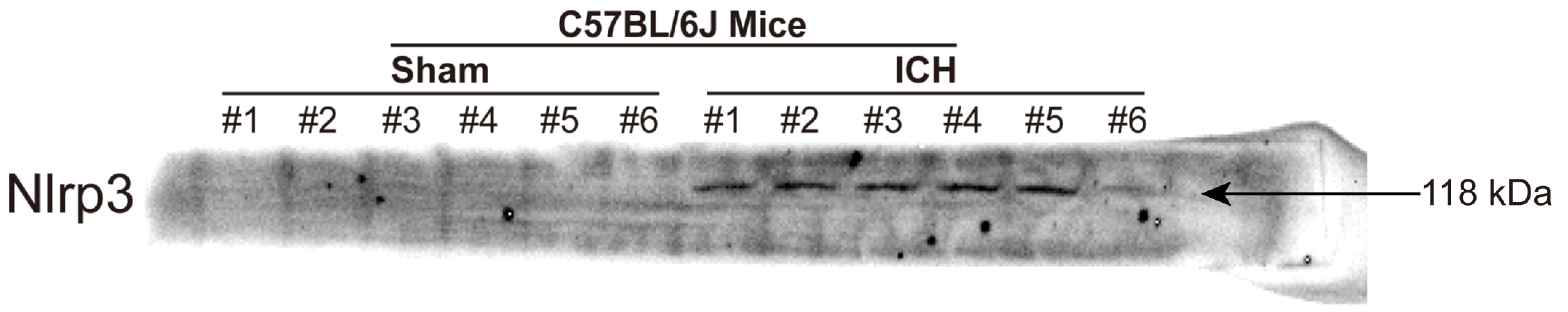

Figure-5H-β-Actin

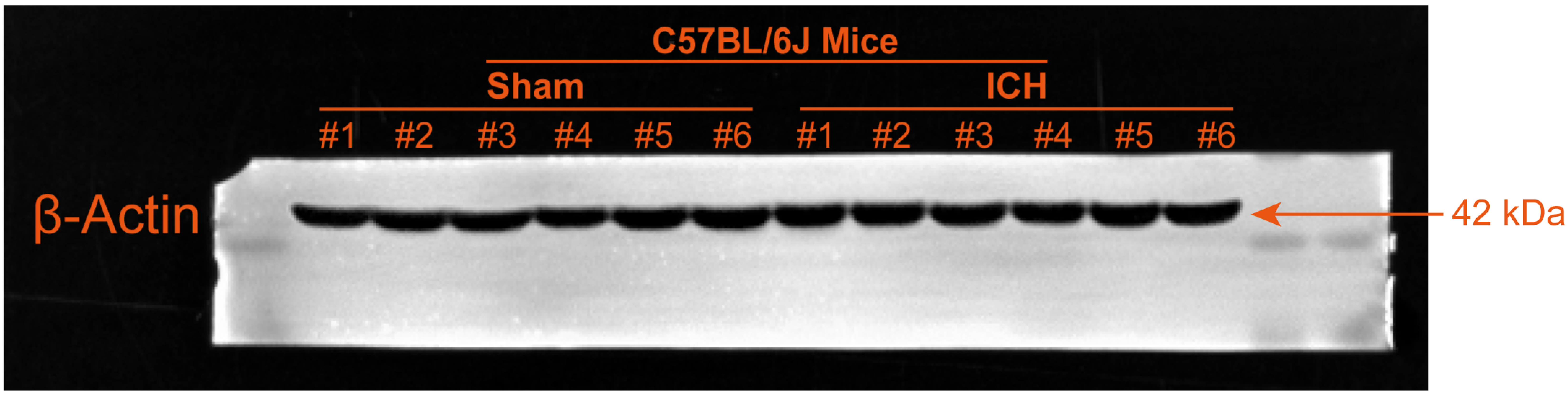

Figure-5K-Nlrp3

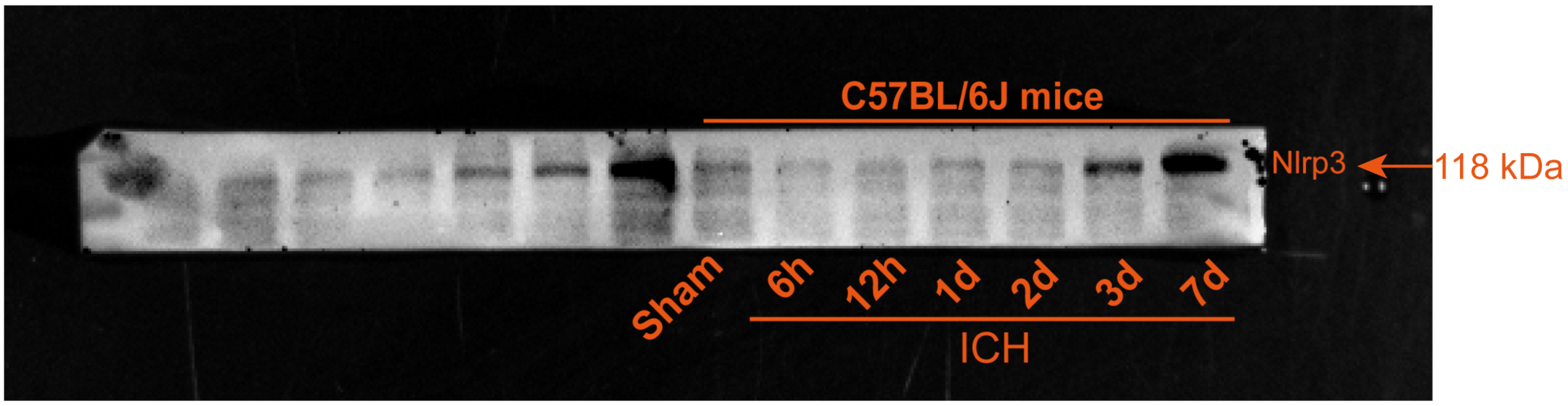

Figure-5K-GAPDH

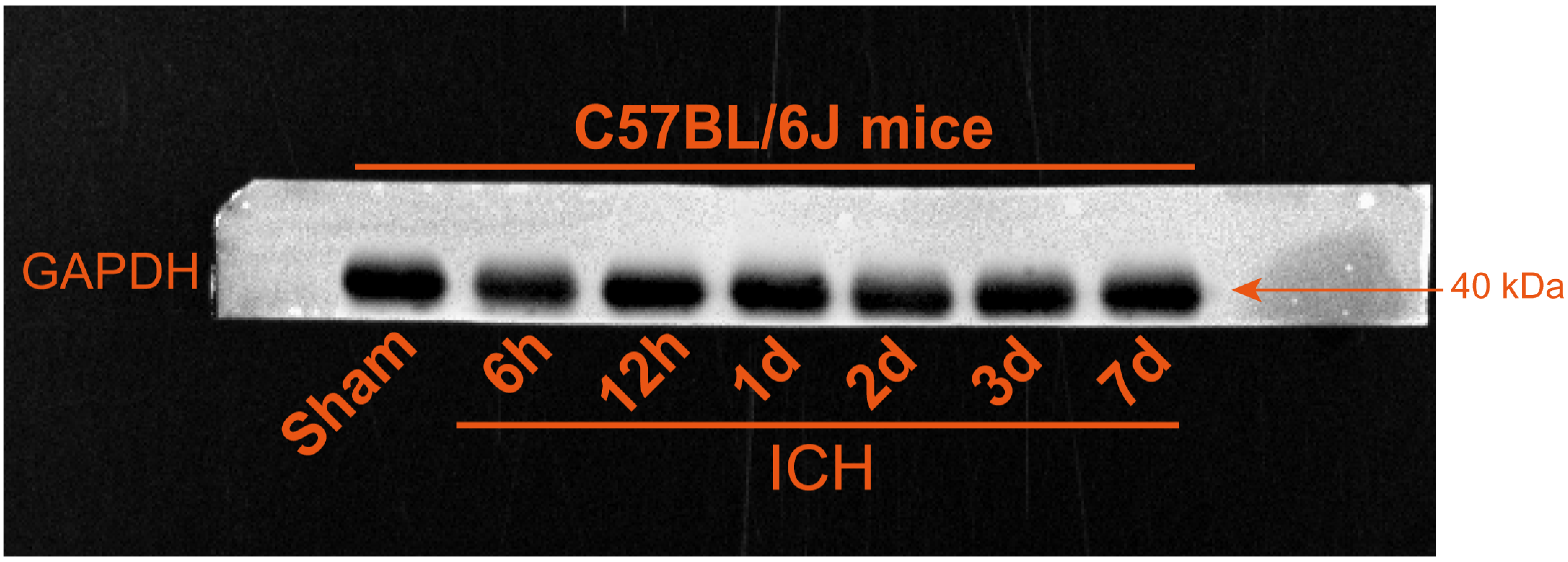

Figure-5P-Sphk1

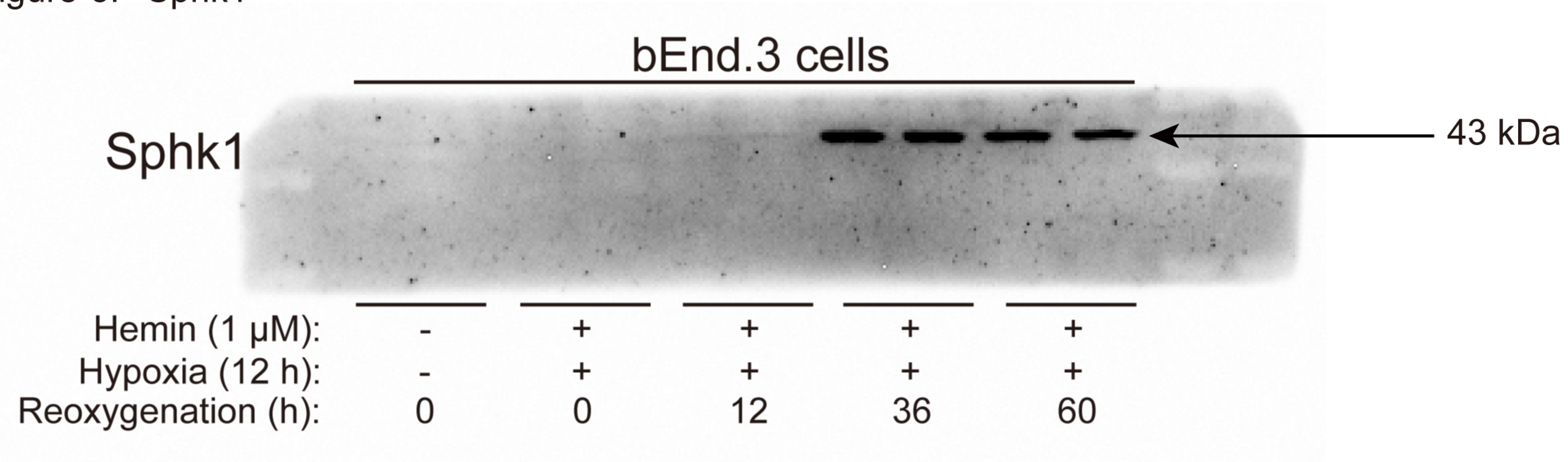

Figure-5P-Nlrp3

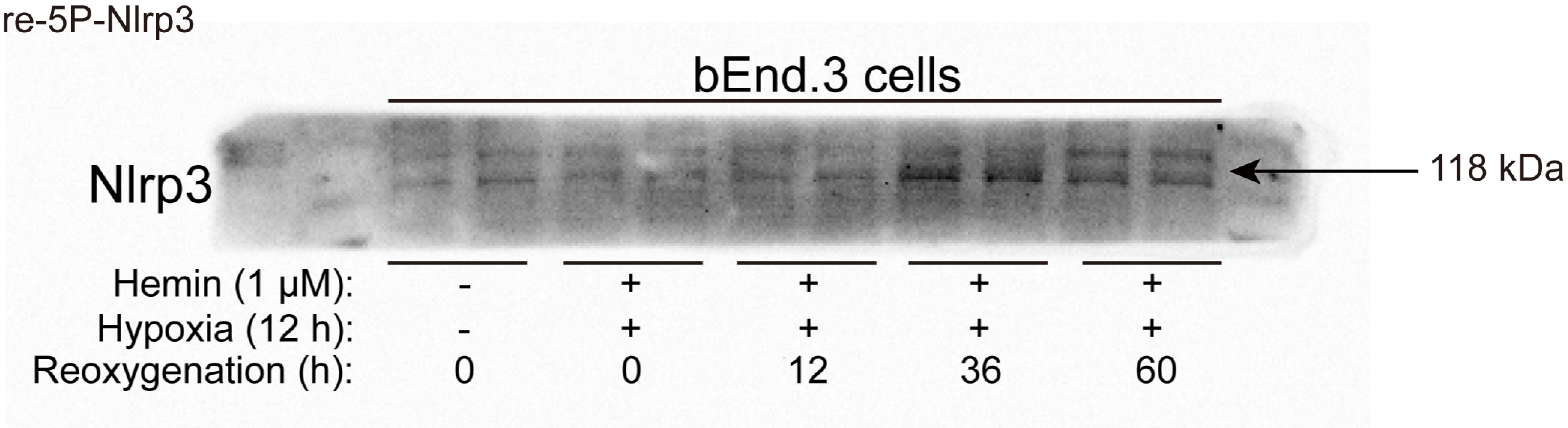

Figure-5P-β-Actin

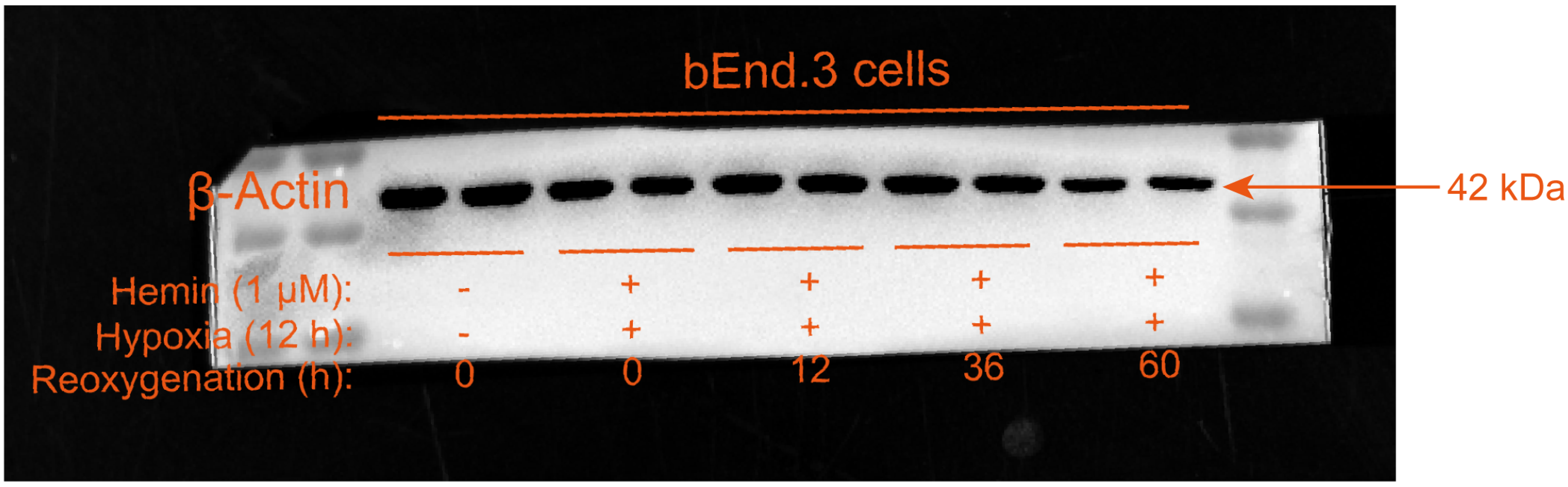

Full uncropped blots images for Figure 6

Figure-6A-Sphk1

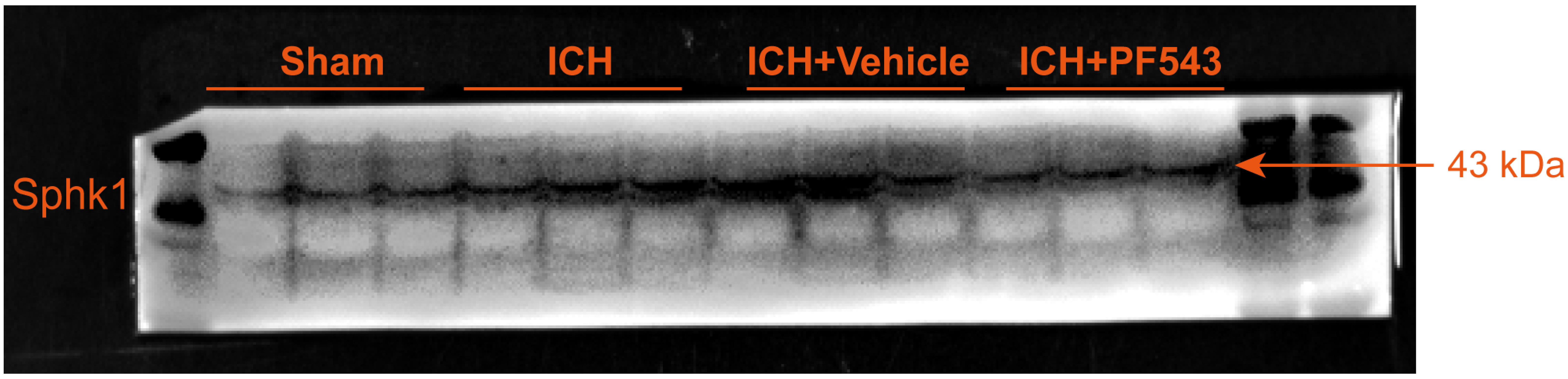

Figure-6A-Nlrp3

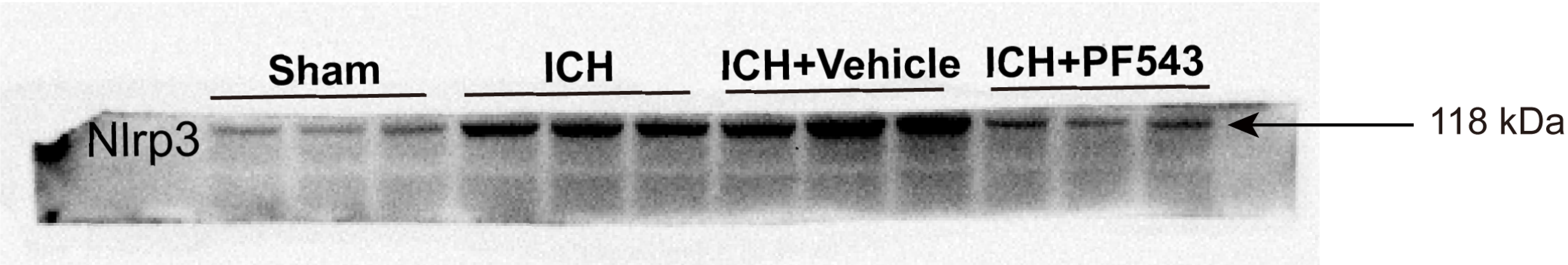

Figure-6A-Caspase-1

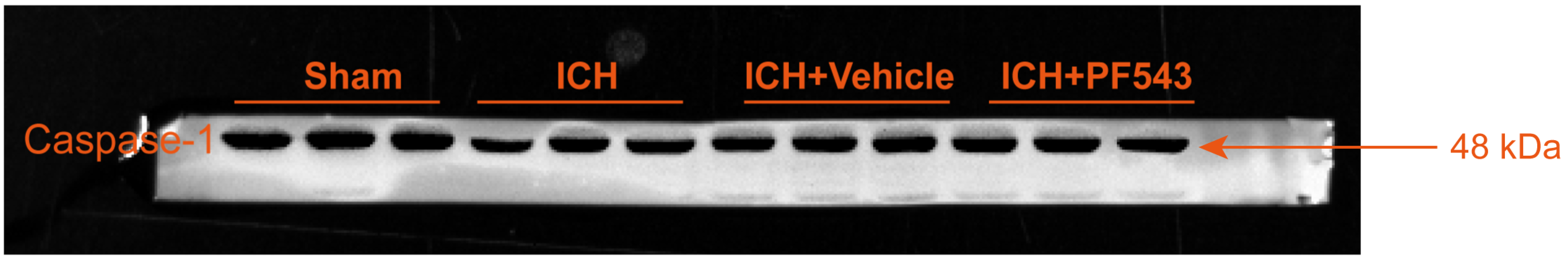

Figure-6A-Cleaved-Caspase-1

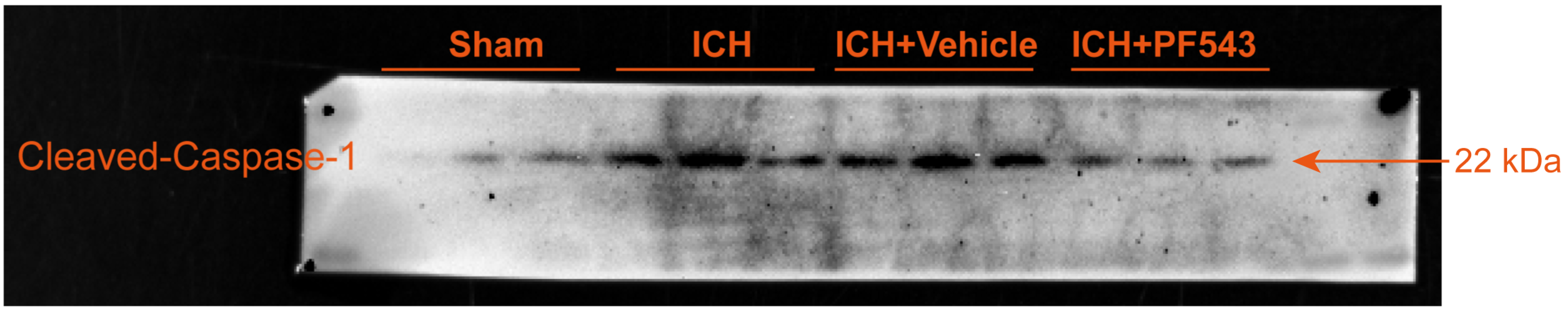

Figure-6A-GSDMD

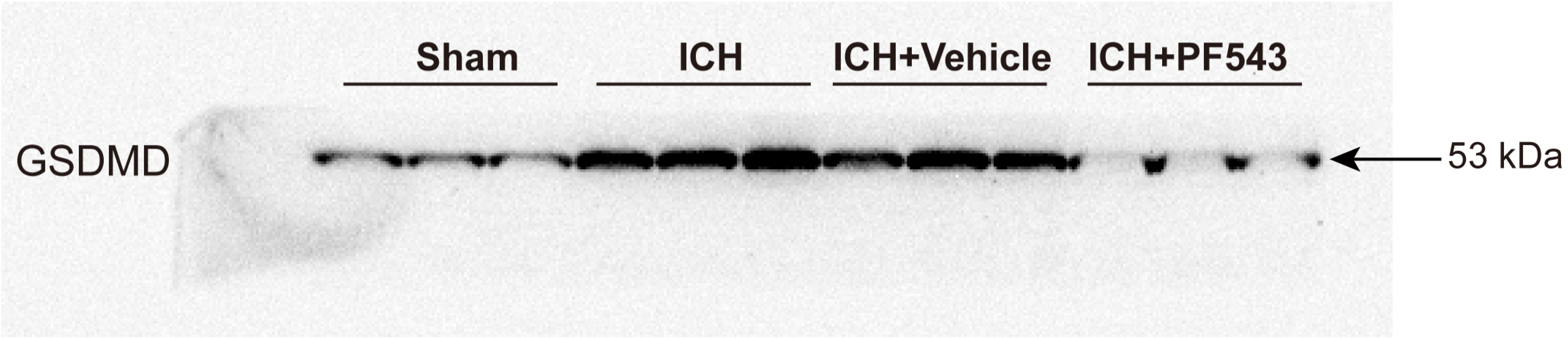

Figure-6A-GSDMD-N

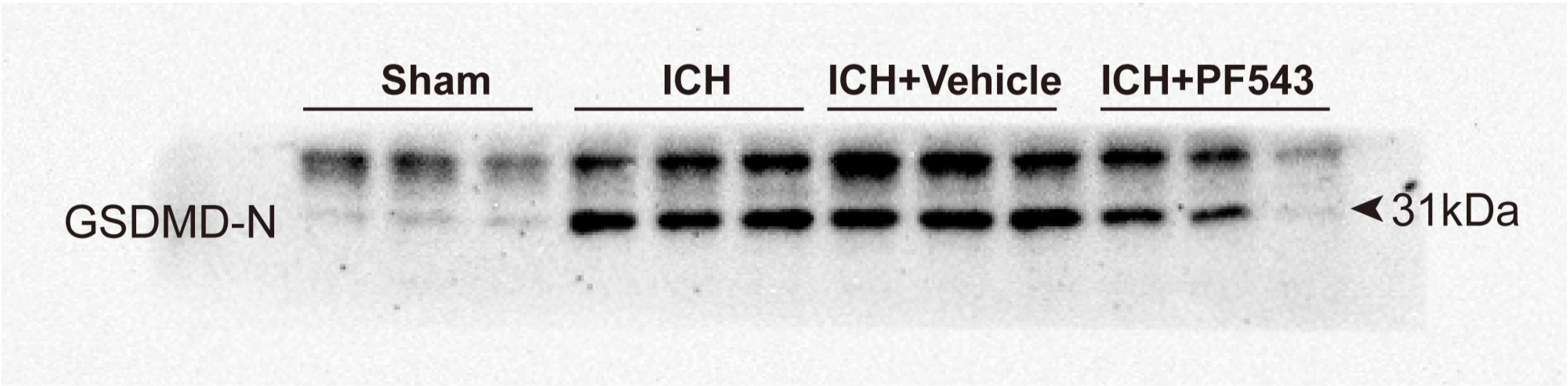

Figure-6A-IL-1β

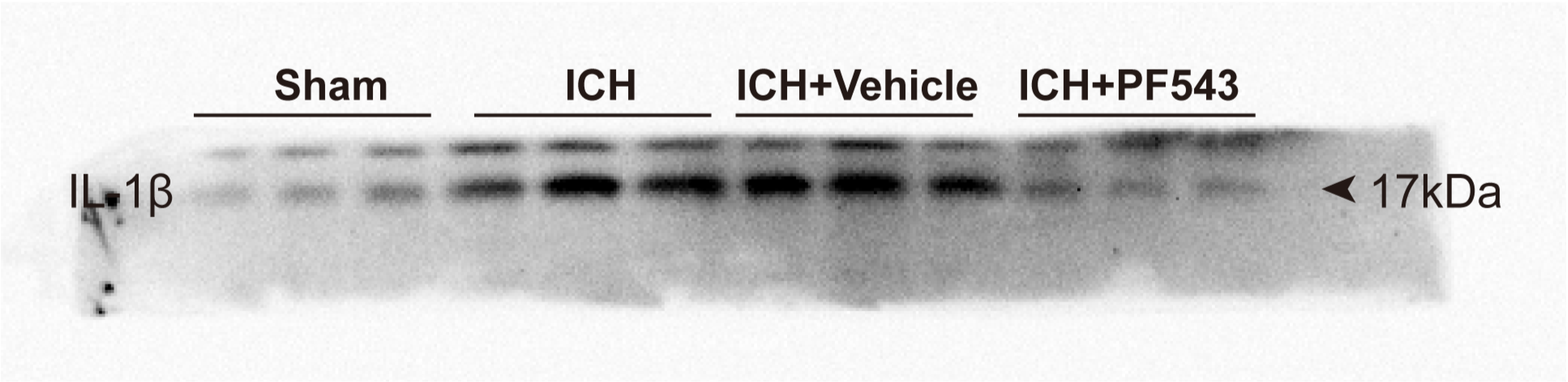

Figure-6A-IL-18

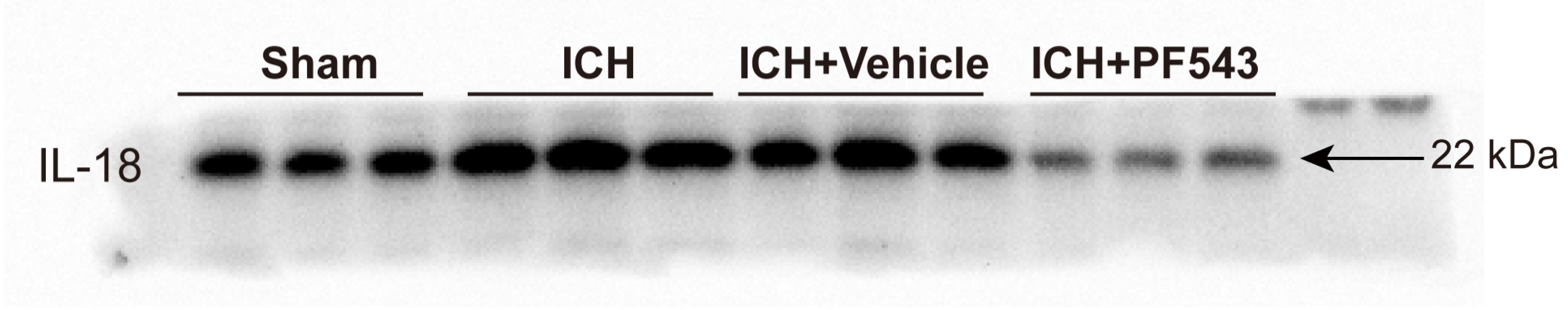

Figure-6A-β-Actin

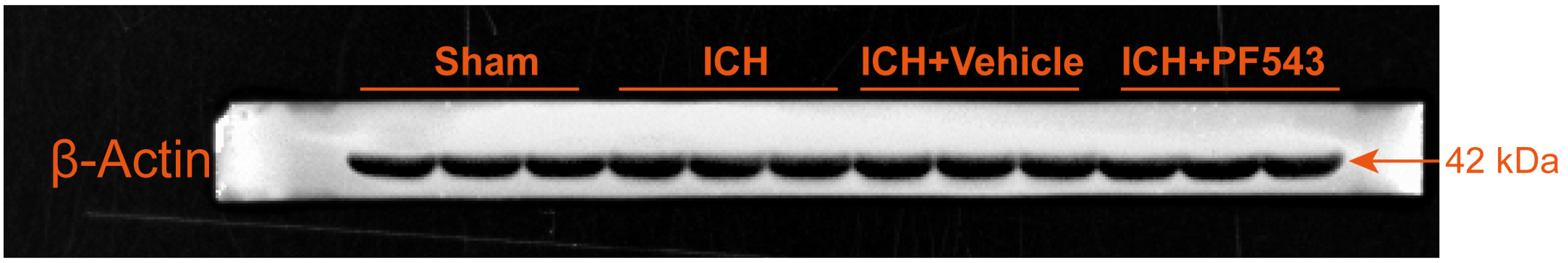

Figure-6N-p-JNK

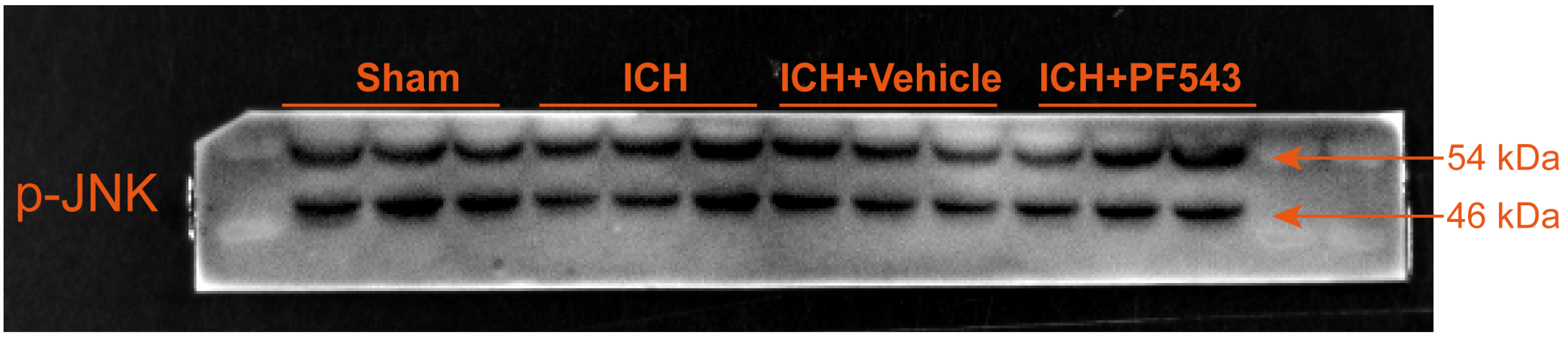

Figure-6N-JNK

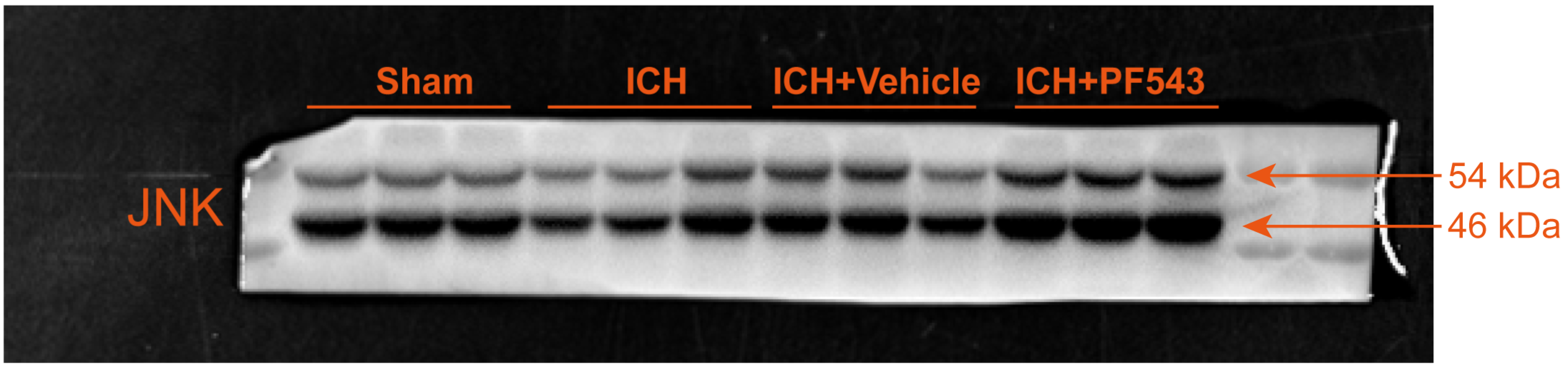

Figure-6N-p-ERK1/2

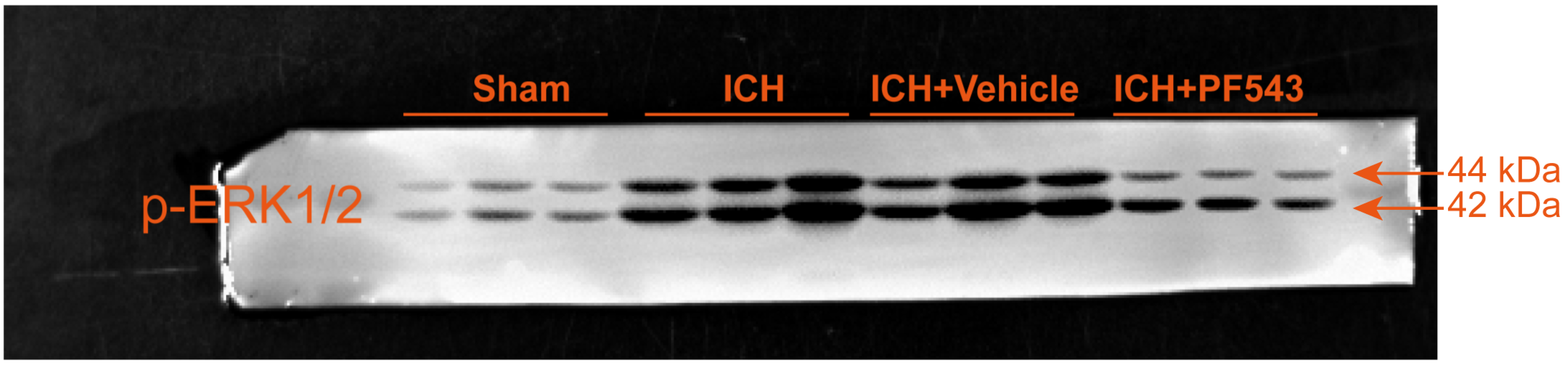

Figure-6N-ERK1/2

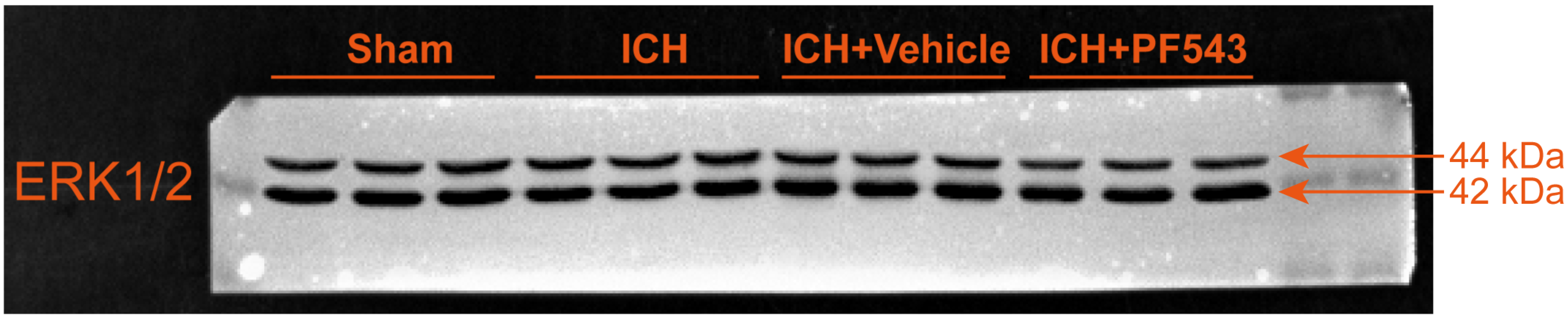

Figure-6N-p-AKT

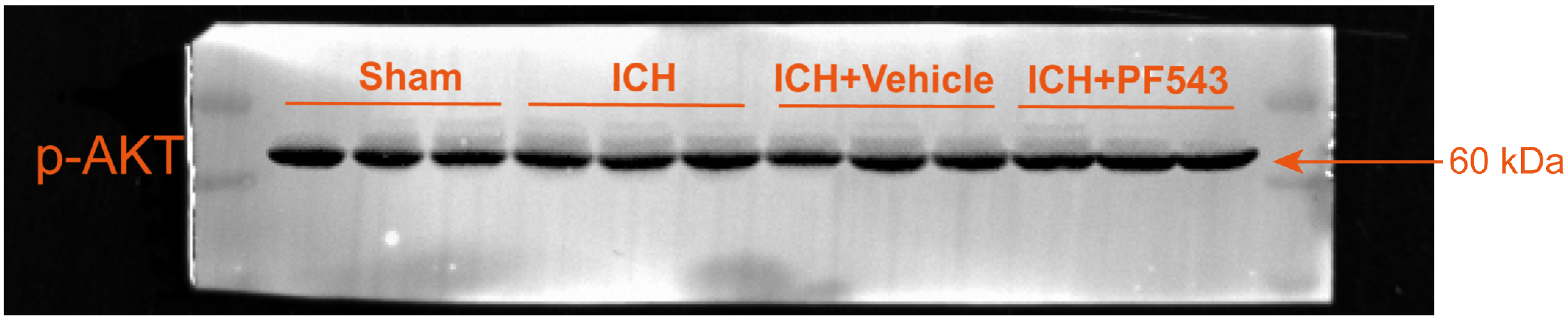

Figure-6N-AKT

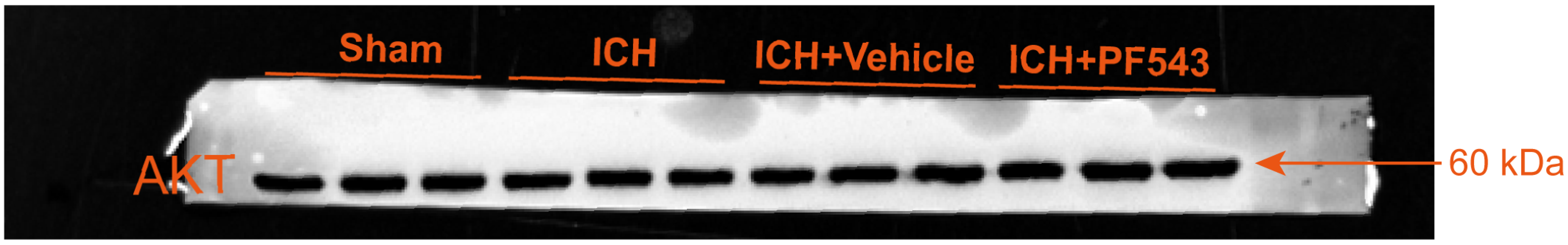

Figure-6N-β-Actin

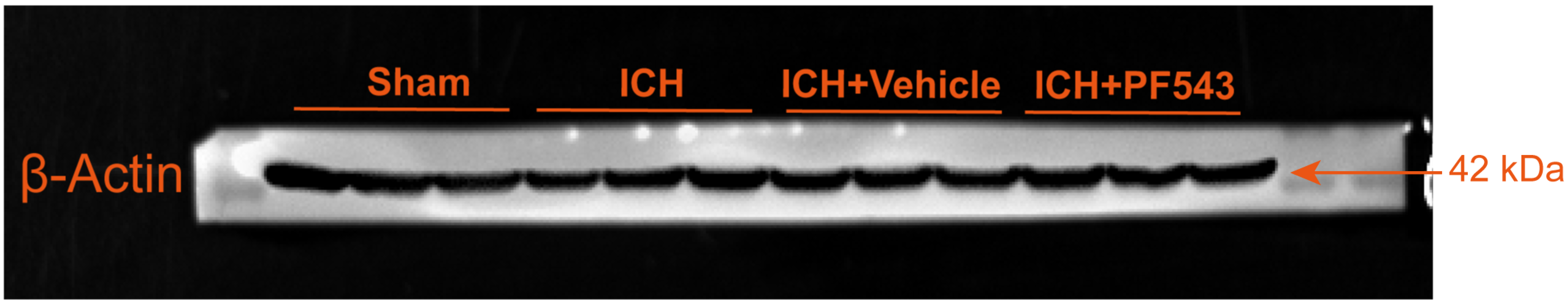

Full uncropped blots images for Figure 7

Figure-7C-Sphk1

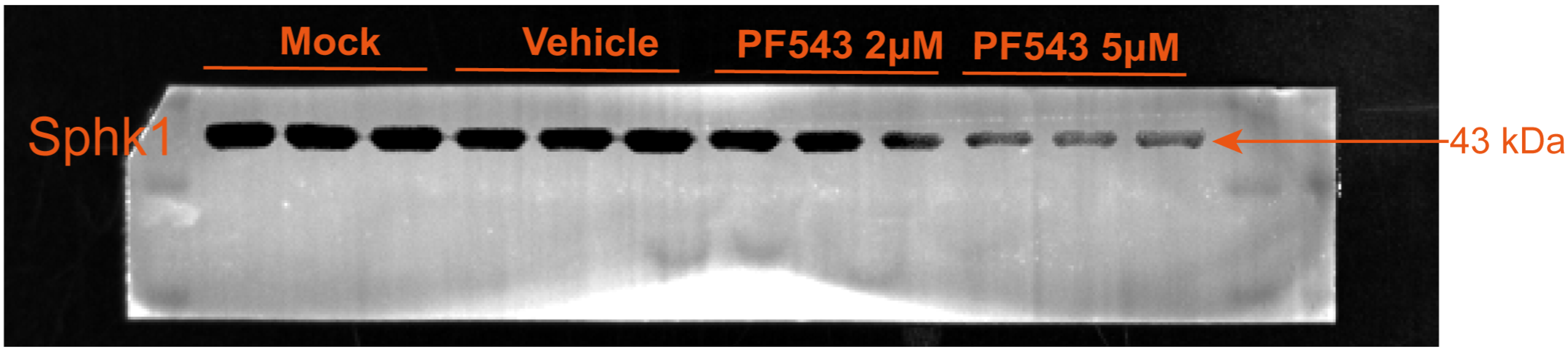

Figure-7C-Nlrp3

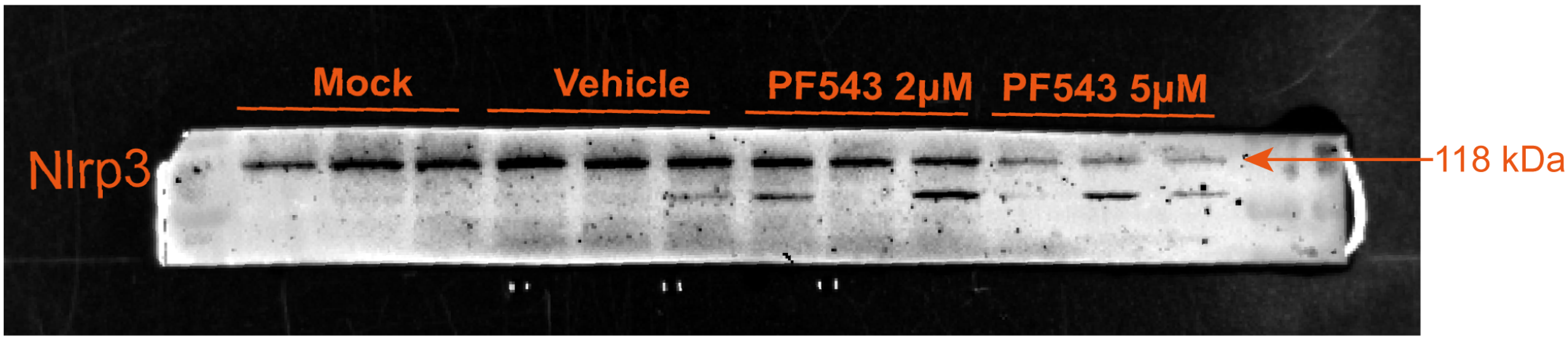

Figure-7C-β-Actin

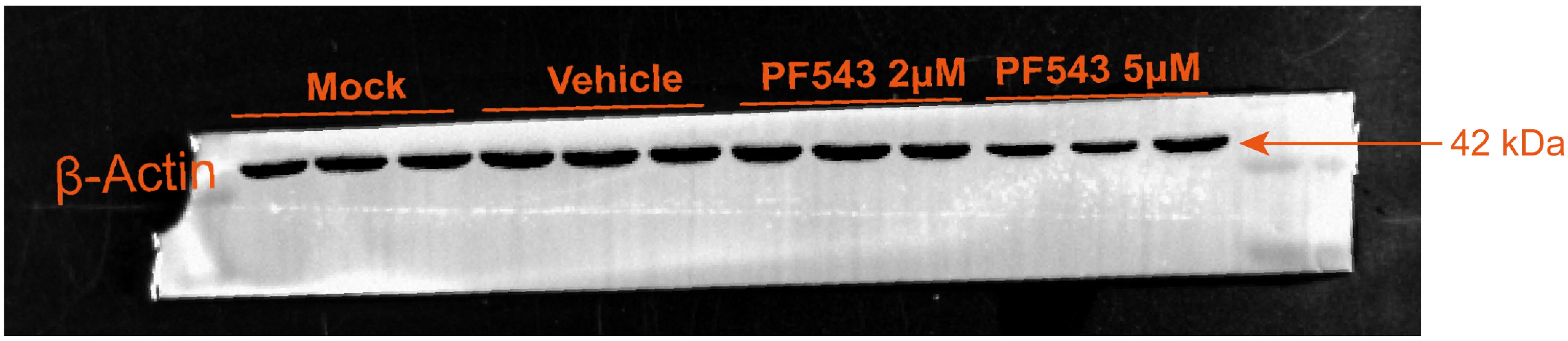

Figure-7K-Sphk1

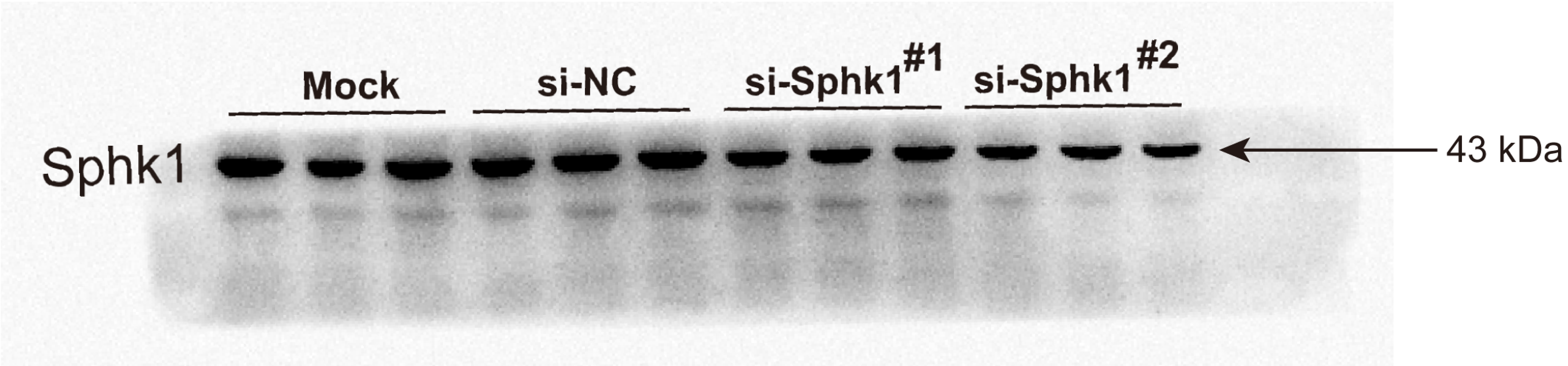

Figure-7K-Nlrp3

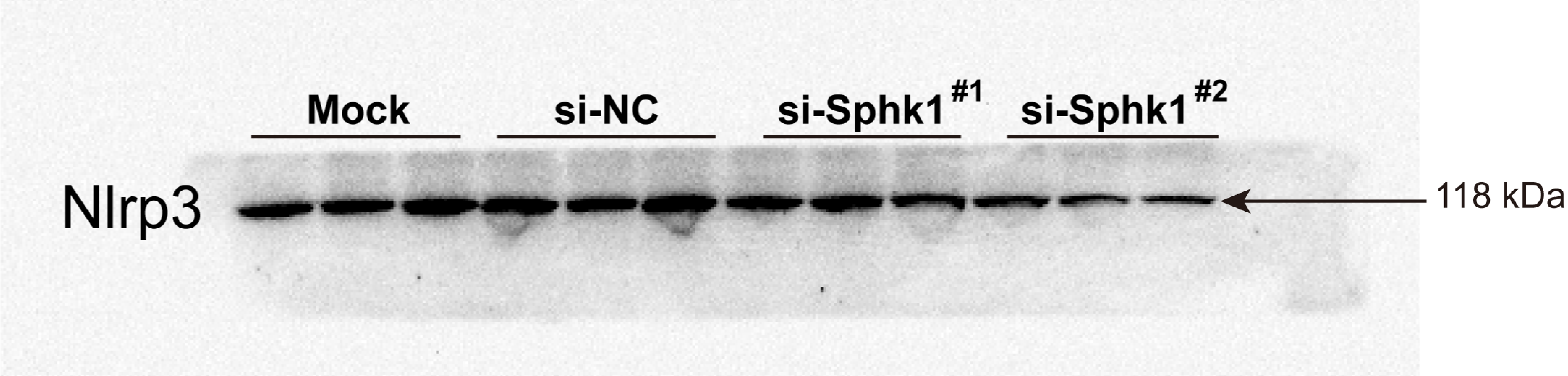

Figure-7K-β-Actin

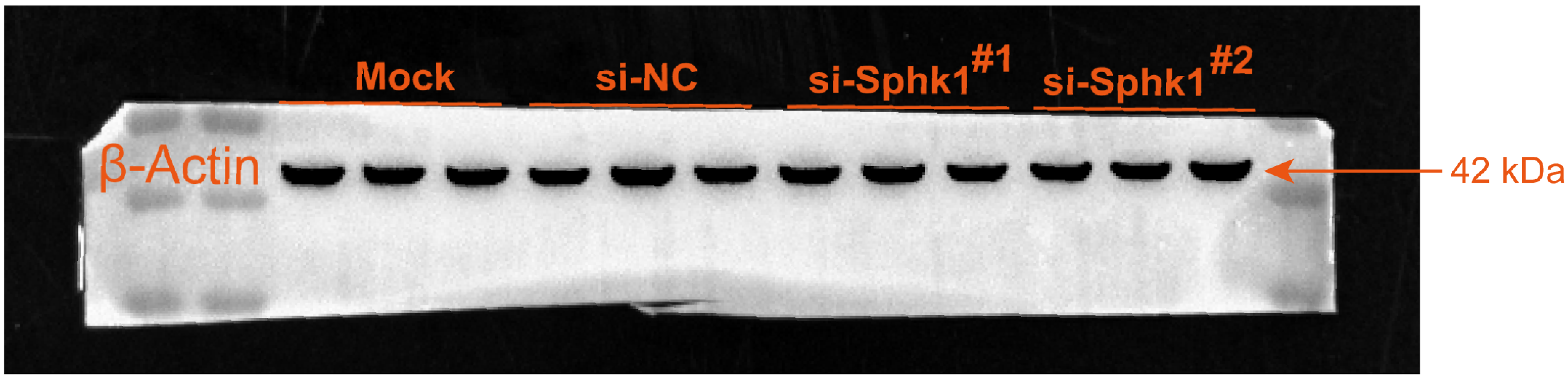

Figure-7R-p-ERK1/2

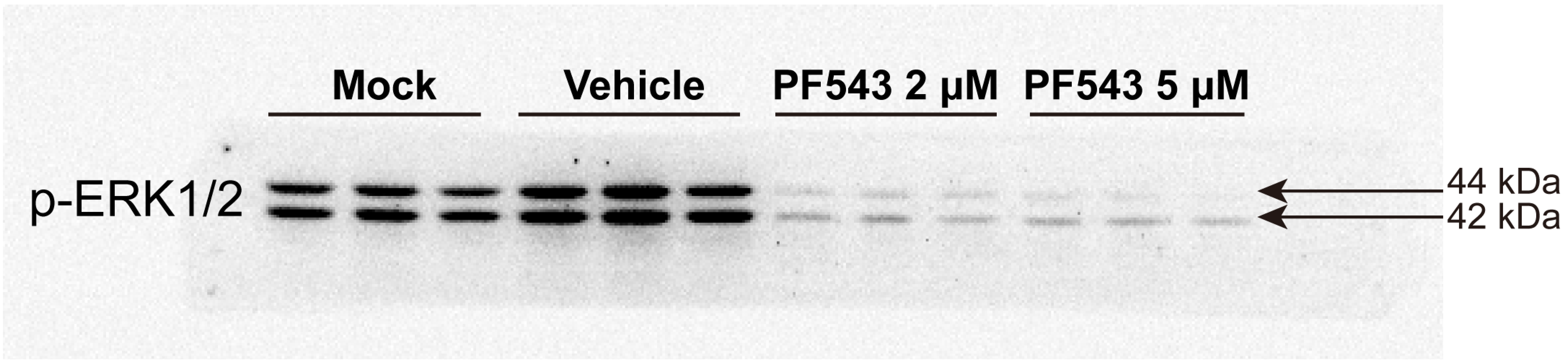

Figure-7R-ERK1/2

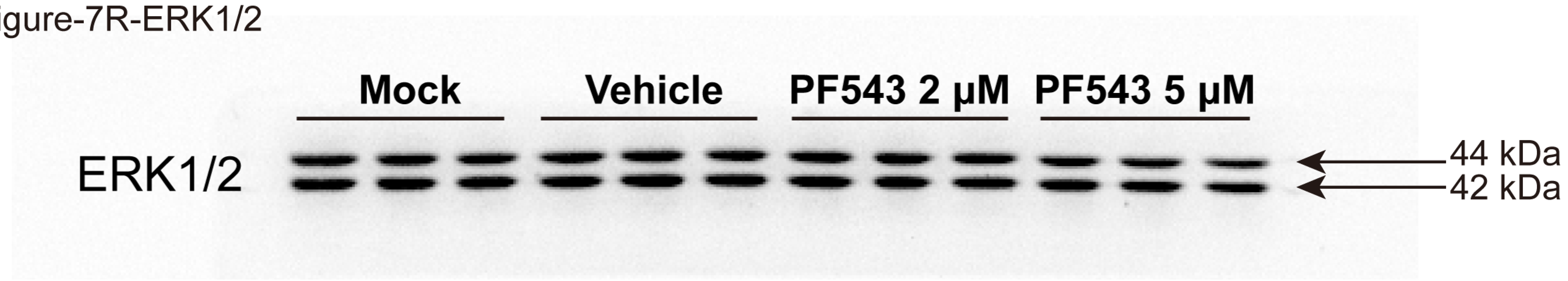

Figure-7R-β-Actin

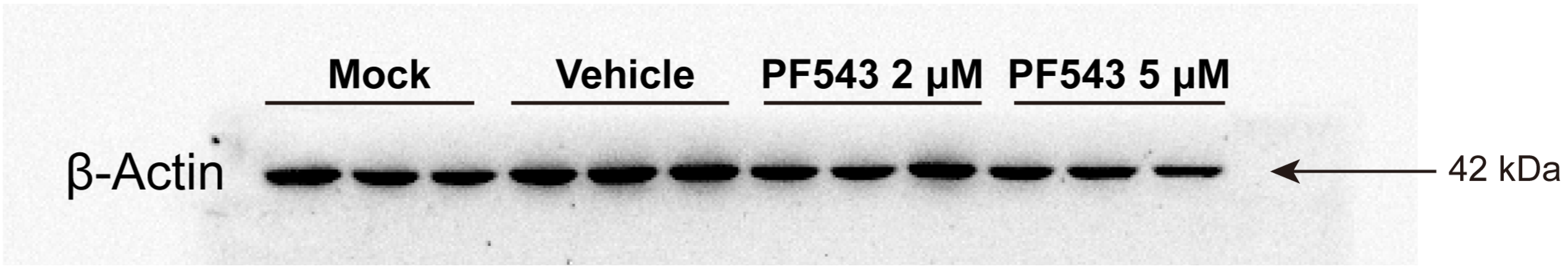

Figure-7T-p-ERK1/2

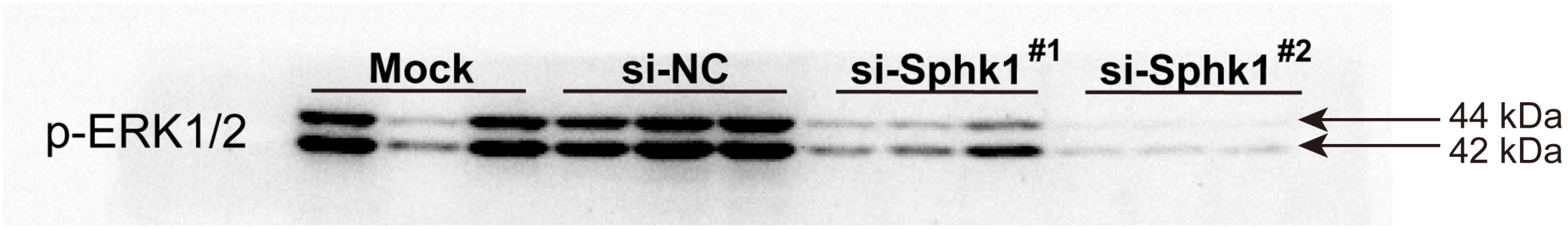

Figure-7T-ERK1/2

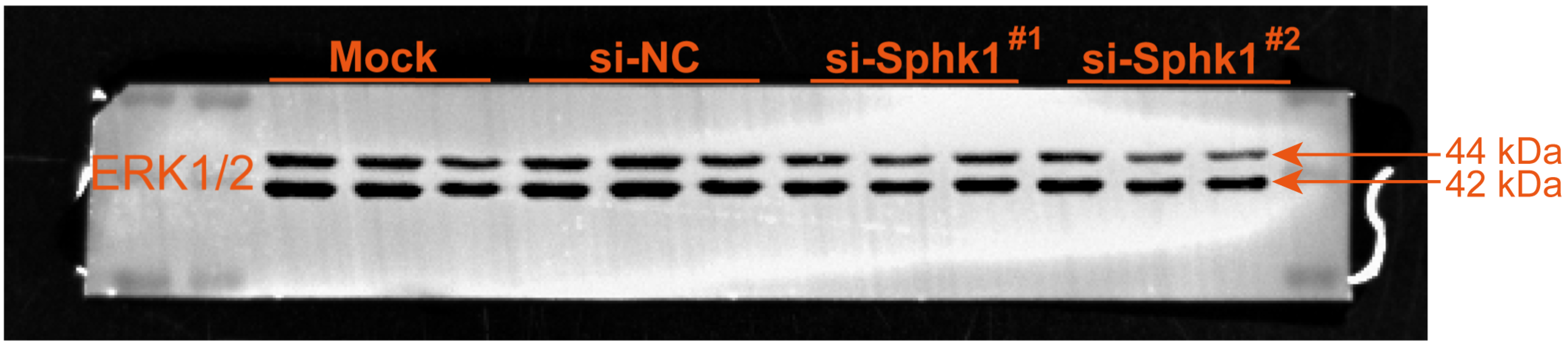

Figure-7T-β-Actin

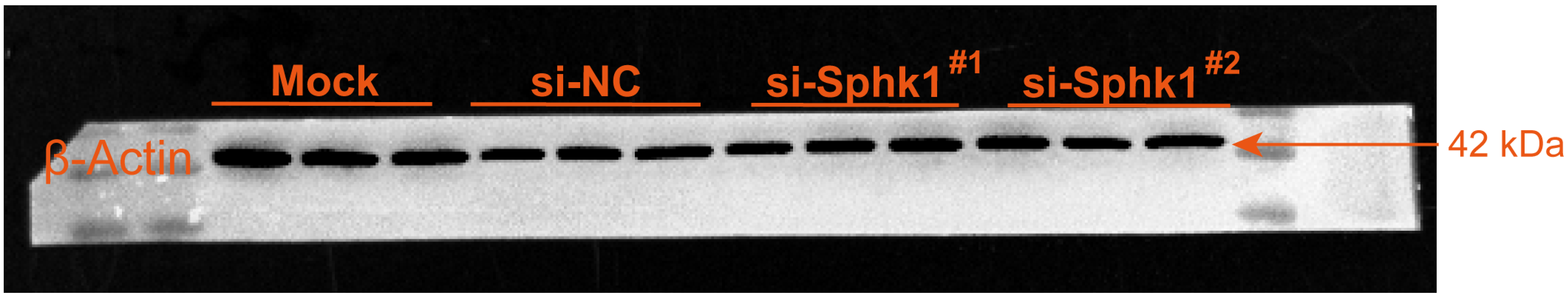

Figure-7X-p-ERK1/2

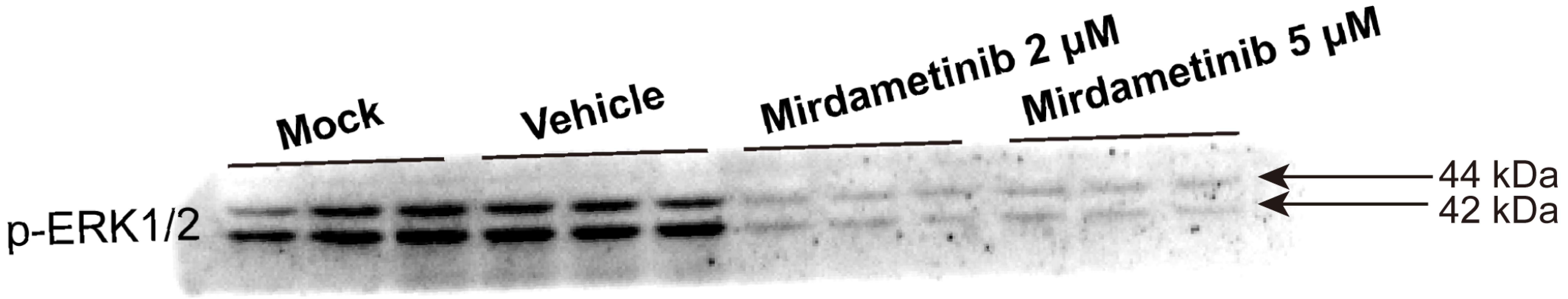

Figure-7X-ERK1/2

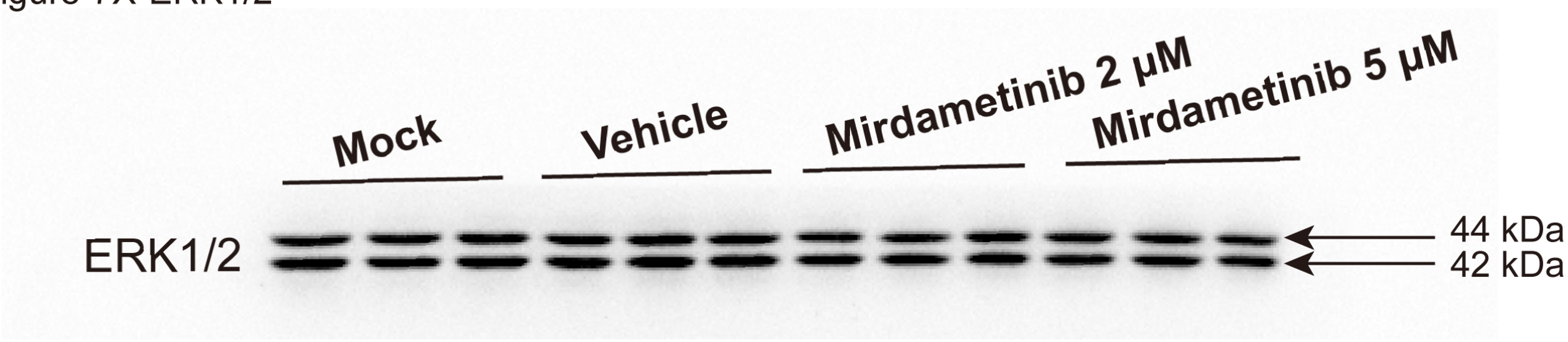

Figure-7X-Sphk1

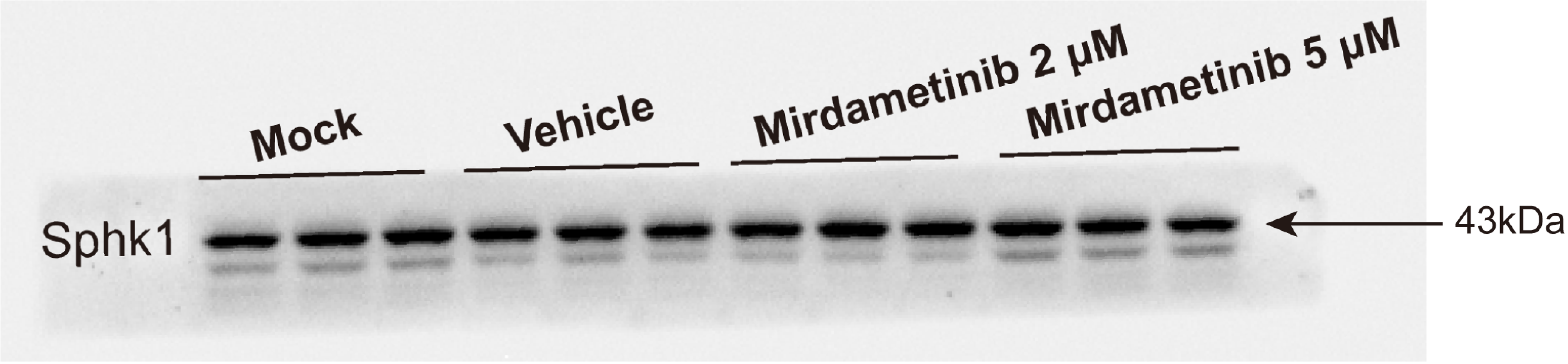

Figure-7X-Nlrp3

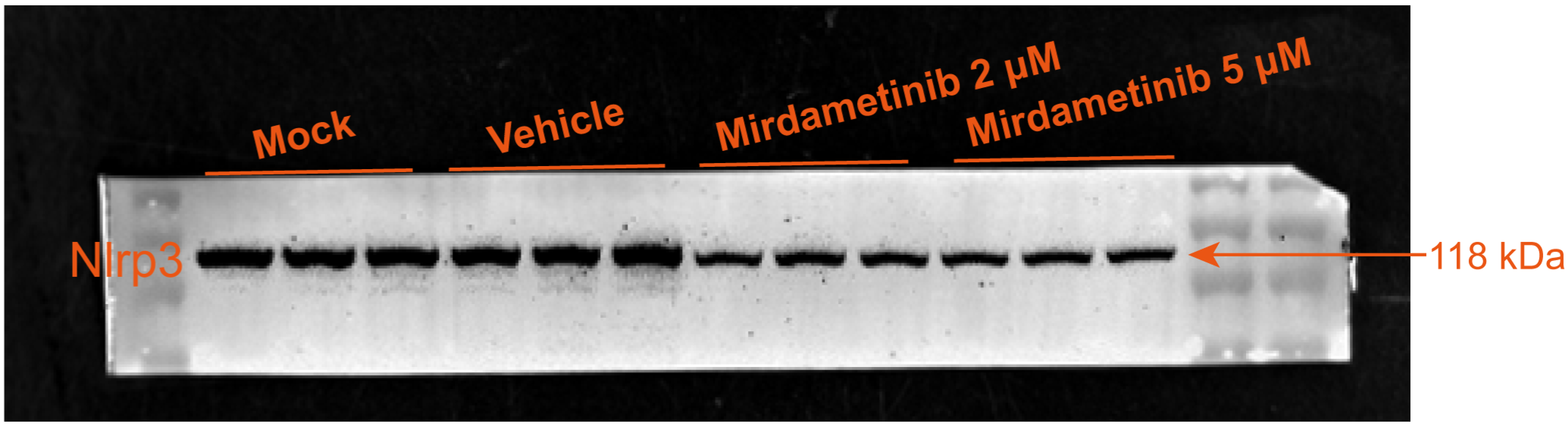

Figure-7X-β-Actin

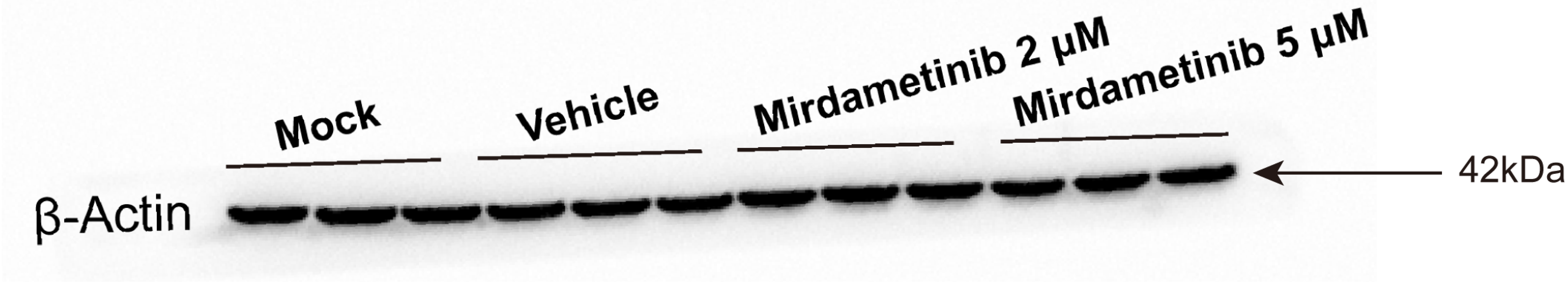

Supplement: Supplementary file 4 — Original WB [file 41419_2024_7310_MOESM4_ESM.pdf]
